# In Situ Process Control during Wet-Chemical Etching of Heteroepitaxial III–V Layers Using Reflection Anisotropy Spectroscopy

**DOI:** 10.1021/acs.jpca.6c02573

**Published:** 2026-07-31

**Authors:** Erica A. Schmitt, Margot Guidat, Marco Flieg, Jonas Grutke, Maximilian Diecke, Kristof Möller, Holger Euchner, Matthias M. May

**Affiliations:** † Institute of Physical and Theoretical Chemistry, Universität Tübingen, Auf der Morgenstelle 15, Tübingen 72076, Germany; ‡ Center for Light-Matter Interaction, Sensors and Analytics LISA+, Universität Tübingen, Auf der Morgenstelle 15, Tübingen 72076, Germany; § Fraunhofer Institute for Solar Energy Systems ISE, Heidenhofstr. 2, Freiburg im Breisgau 79110, Germany; ∥ AZUR SPACE Solar Power GmbH, Theresienstraße 2, Heilbronn 74072, Germany

## Abstract

The preparation of semiconductor surfaces for renewable energy or other optoelectronic applications requires highly precise control of surface structure and composition, rendering vacuum-based techniques typically the method of choice. However, when precise process control is guaranteed, wet-chemical etching processes constitute a resource-efficient and scalable alternative. In this work, we show that reflection anisotropy spectroscopy (RAS)—a nondestructive, optical technique—offers a powerful in situ control of wet-chemical etching processes. Here, we employ RAS to determine the etching depth, rate, and time during the wet-chemical preparation of a photoelectrode for direct solar water splitting. This is achieved by observing and evaluating Fabry–Pérot-type oscillations of reflectance and RAS transients during the etching process. The occurrence of these oscillations confirms the uniform character of the investigated surface in a suitable etching parameter space and allows the determination of the etching depth with a precision of ±8 nm. A variation of the etching procedure demonstrates that RAS control can be used for the development of suitable etching routines and for in situ process control, as validated for two different types of photoelectrodes.

## Graphical abstract

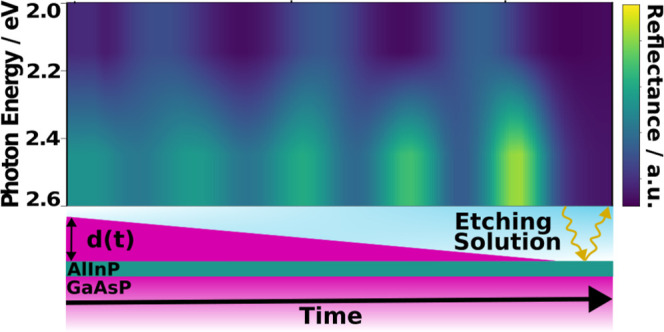

## Introduction

For renewable energy transport and storage such as for the production of carbon-free fuels, electrochemical and wet-chemical processing play a key role. Improving efficiency and applicability of corresponding sustainable technologies therefore requires a good microscopic understanding of wet-chemical reactions on complex heterocatalytic surfaces and electrochemical interfaces.^1–3^

The dynamic nature of (photo)­electrode surfaces undergoing structural changes under operation highlights the need for in situ methods.^4^ Only a few of such operando characterization techniques, which can convey information about heterocatalytic reactions at the electrode–electrolyte interface, are currently available. However, these are often limited in number and application range with respect to the in situ observation of (electrochemical) reactions in the wet-chemical environment, especially with regard to process control, for instance, in the case of electrochemical surface functionalization. Typical reasons include the need for extensive instrumentation, long data acquisition times, or a destructive nature of the method.

Precise wet-chemical modification in (electro)­chemical environments is highly relevant for semiconductor fabrication, also in applications beyond photoelectrochemistry, such as photovoltaics.^5,6^ Specifically, the transformation of epitaxially grown III–V semiconductor-based multijunction solar cells into photoelectrodes for hydrogen production is examined in the following. In photoelectrochemical (PEC) water splitting, the solar cell front and backside are employed as photocathode and -anode, respectively, with the solar cell being immersed in an acidic electrolyte.^7,8^ The semiconductor–electrolyte interface works here as a charge-selective contact layer which reduces charge-carrier recombination and acts as an (electro)­chemical passivation layer.^9^ Passivation is necessary to protect the immersed photocathode from corrosion, which is currently a bottleneck for the stability and therefore the upscaling of PEC devices.^10,11^ Precise pretreatment of solar cells and the control thereof is hereby critical to create a homogeneous surface for subsequently deposited, oxide-based passivation layers.

Here, reflection anisotropy spectroscopy (RAS), a nondestructive optical technique is well-suited, as it offers the opportunity to observe electrochemical and wet-chemical processes at the very electrode–electrolyte interface.^12^ Monitoring changes in RA spectra enables, e.g., the observation of solar cell growth in metal organic chemical vapor deposition (MOCVD) processes.^13^ For the control of epitaxial growth in MOCVD processes, RAS monitoring is established commercially.^14^ Reversing the perspective from growth to material removal, the works of Kleinschmidt and Sombrio et al.^13,15^ show the ability of RAS to give detailed information about dry etching processes, including precise determination of etching time, rate, and depth. In their works, the occurrence of Fabry–Pérot oscillations in the RA spectra is used to determine these quantities.

In principle, this is also possible for controlled wet-chemical etching of monocrystalline materials. Recently, the in situ control of etching processes with RAS was applied in the preparation of photoelectrochemical (PEC) cells for direct water splitting.^8^ This work aims to extend the approach to the determination of etching depth and rate by interpreting Fabry–Pérot oscillations of reflectance transient measurements during wet-chemical etching. To our best knowledge, this is shown here for the first time. The correlation of RA spectra with subsequent X-ray photoelectron spectroscopy (XPS) and atomic force microscopy (AFM) investigations furthermore aims at validating RAS and reflectance oscillations as evidence for the uniform nature of the etching process. At the same time, RAS features that originate from surface roughening are identified.^16,17^

In this work, we showcase the use of Fabry–Pérot oscillations in RA and reflectance transients and spectra to control the removal of a protective, 250 nm GaAsP cap layer from a multijunction III–V/Si solar cell, as schematically depicted in Figure 1. The solar cells used here are directly epitaxially grown on a Si substrate, using a step-graded metamorphic buffer structure to overcome the lattice mismatch between silicon and the III–V absorber layers. While the Si or the buffer layers are located far away from the surface of the solar cell and therefore not etched here, they might have an indirect impact on the etching process due to threading dislocations present in the III–V layers originating from relaxation of the crystal lattice during growth. The in situ etch depth monitoring facilitates a precise and controlled removal of the GaAsP cap layer from the heterojunction solar cell. Moreover, the occurrence of oscillations in transient measurements allows us to evaluate the homogeneity of the etching procedure in situ, thus serving as quality control.

**Figure 1:**
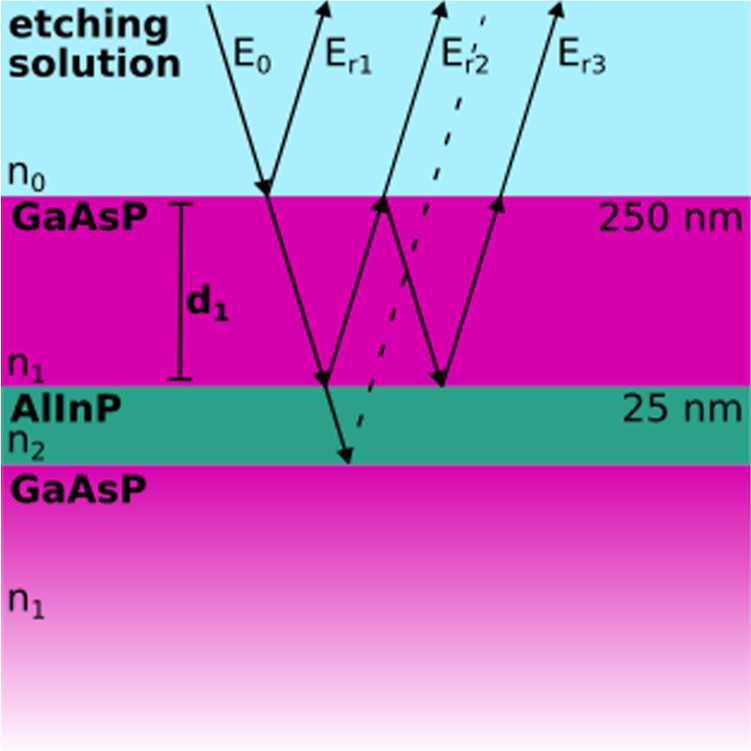
Cross-section of a III–V multijunction solar cell immersed in an etching solution with refractive index *n*_0_, the GaAsP cap layer (*n*_1_), AlInP window layer (*n*_2_), and GaAsP top absorber (*n*_1_). The thickness of the cap layer *d*_1_ is decreasing over time by wet-chemical etching. The impinging light beam *E*_0_ is partially reflected at the respective material interfaces, leading to time-dependent interference of the reflected light beams *E*_rn_. This results in Fabry–Pérot oscillations of the reflectance and the RA signal.

## Methods

### Reflection Anisotropy Spectroscopy

RAS uses linearly polarized white light at near-normal incidence to probe the optical response of a given sample. To obtain the RA spectrum, the difference in reflectivity between two orthogonal directions of an anisotropic single-crystal surface is determined as a function of the photon energy and normalized to the overall reflectivity. This corresponds to dividing the difference in the complex eigenvalues r_*x*_ and r_*y*_ of the Fresnel reflection coefficients along the two orthogonal axes x and y by their average value, as given in eq 1.^18^ In experiment, typically only the real part is plotted.

1 $$RAS≔R\left(\frac{\Delta r}{r}\right)=R\left(2\times \frac{r_{x}-r_{y}}{r_{x}+r_{y}}\right);r\in C$$

RAS combines a time resolution in the order of milliseconds for commercial spectrometers with a surface sensitivity of submonolayer scale.^19^ The interface-sensitivity with respect to atomic ordering, combined with sensitivity toward adsorbates, makes it possible to quantitatively resolve surface structures in the submonolayer range. Hereby, the optical response is averaged over the area probed by the light spot, typically having a size of about 0.5 cm^2^. RAS is operable in liquid electrolytes with sufficient transparency in the optical regime, therefore making it also suitable for in situ electrochemical process control.^20^ In addition to the RA signal, the reflectance R = |*r* ^2^|, corresponding to the real square of the Fresnel reflection amplitude, is necessarily probed and recorded in parallel. In this work, the detector’s voltage referred to as “DC signal”, which is directly proportional to the reflectance, is reported (see Supporting Information Section S2.1). In the following, “DC signal” and “reflectance” are used synonymously, neglecting a normalization with respect to a reference.

The RA spectra were measured with an RA spectrometer (EpiRAS from Laytec) equipped with a Xenon light source. The setup used for the in situ etching monitoring is illustrated elsewhere and in the Supporting Information.^8^ To allow RAS measurements of the surface of the photoabsorber mounted vertically in the glass cell, a mirror is placed below the spectrometer at an angle of 45° and in front of the sample. The light, initially linearly polarized, is reflected on the mirror and reaches the sample. An eventual imperfect vertical position of the sample is corrected by an antiwobble mirror (AWM) subsequent to the first reflection of the light on the surface. After the second reflection on the photoelectrode, the light, now elliptically polarized due to the optical anisotropy, reaches the detector of the spectrometer.

The reference spectrum of the epitaxial layer stack, corresponding to the GaAsP-terminated surface, was taken in water during the cleaning process of the cell. For monitoring the etching, a solution of H_2_O_2_/NH_4_OH/H_2_O (H_2_O_2_ supplied by Sigma-Aldrich, p.a., 30%, NH_4_OH supplied by VWR, p.a., 25%) was prepared, with different volume ratios. Before starting the transient measurement, the PEC cell was filled with the etching solution just below the bottom of the electrode. 10 s after the start of the transient, the electrode was fully covered with the etching solution. To prevent the etching of AlInP by the residual etching solution present on the surface of the electrode, the PEC cell was rinsed three times with ultrapure water (Milli-Q grade) within 1 min after the etching process was completed. The spectrum after etching was measured in water during the third cleaning step. Note that for all spectra, a baseline correction is applied to the RA spectra and to the transient by subtracting the sum of two spectra, where the sample has been rotated by 90° in between.

### Sample Preparation

An organic protective coating applied to the samples described in Figure 1 for wafer sawing and transport is removed by cleaning each sample twice in acetone (Sigma-Aldrich, HPLC grade), once in isopropanol (VWR, HPLC grade), and once in water (Milli-Q grade). The samples C0–C4 and C8 are analyzed with RAS, XPS, and AFM (see Supporting Information Section S3.5). These samples and C7 were mounted on a glue-free sample holder using a clamping mechanism (see Supporting Information Figure S1). The sample is etched in a Schlenk cell that is flushed with N_2_ during the cleaning procedure and cleaned three times with water (Milli-Q grade). RAS reference spectra were measured during the third cleaning step. C5 and C6, analyzed by RAS only, were glued from the back side to a glass rod with epoxy glue and electrically contacted with a silver resin.

The second type of samples, provided by AZUR SPACE, (see Figure 6) are based on their “TJ Solar Cell 3G30C—Advanced” epitaxial structure with a bottom-up monocrystalline layer stack of Ge (band gap 0.7 eV), GaAs (1.4 eV), and GaInP (1.8 eV) and a 500 nm GaAs cap layer and measured analogously.

**Figure 2:**
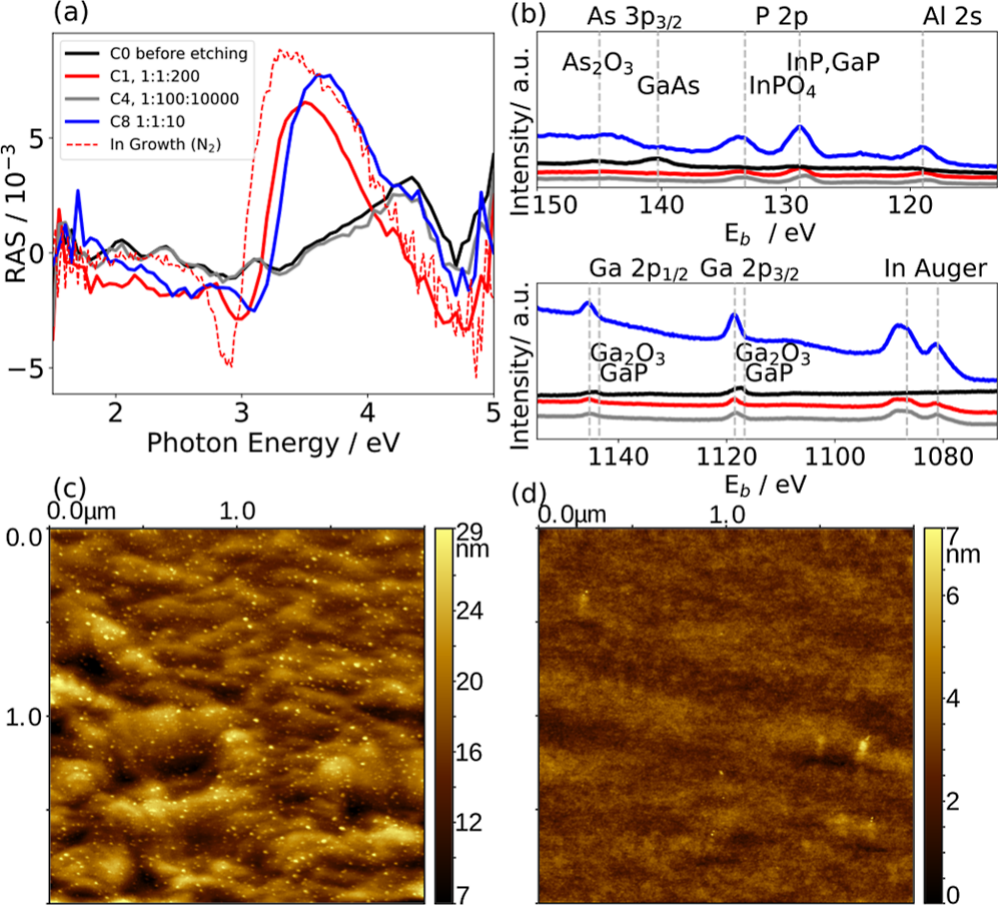
(a) RA spectra of GaAsP-based samples C0 before etching, as well as C1, C4, and C8 after etching. For comparison with the etched solar cells, the RA spectrum of the AlInP window layer during growth (ex situ, N_2_ atmosphere) is depicted in dashed lines. (b) XPS analysis of In Auger, As 3p, P 2p, Al 2s, and Ga 2p core levels for samples C0, as well as C1, C4, and C8. AFM images of (c) C0 (before etching, rms roughness = 3.2 nm) and (d) C1 (after etching, rms roughness = 0.6 nm).

**Figure 3:**
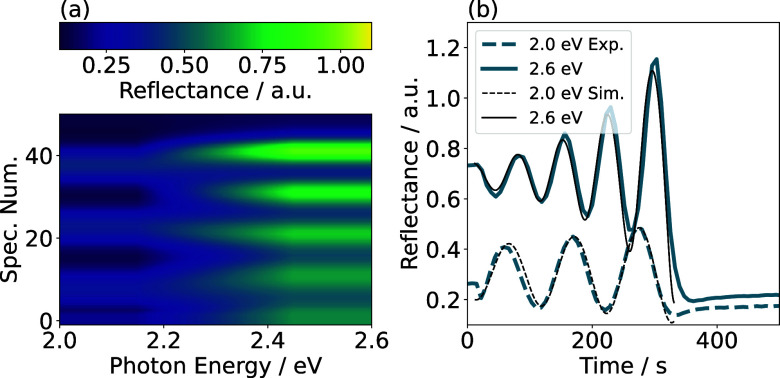
(a) CP of the reflectance signal acquired upon etching of GaAsP-based sample C1 at 2.0–2.6 eV. (b) Time-resolved reflectance signals extracted at 2.0 and 2.6 eV from the respective CP. The black lines show the respective simulated oscillations for the given layer stack (see eqs 2–7).

**Figure 4:**
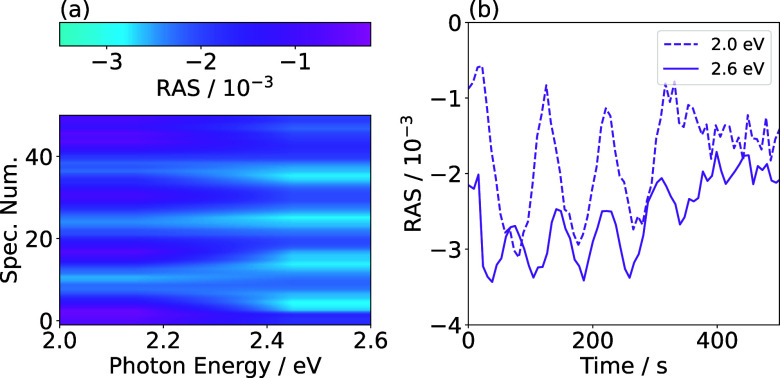
(a) CP of the RA spectra acquired upon etching of GaAsP-based sample C1 at 2.0–2.6 eV. (b) Time-resolved reflectance signals extracted at 2.0 and 2.6 eV from the respective CP.

**Figure 5:**
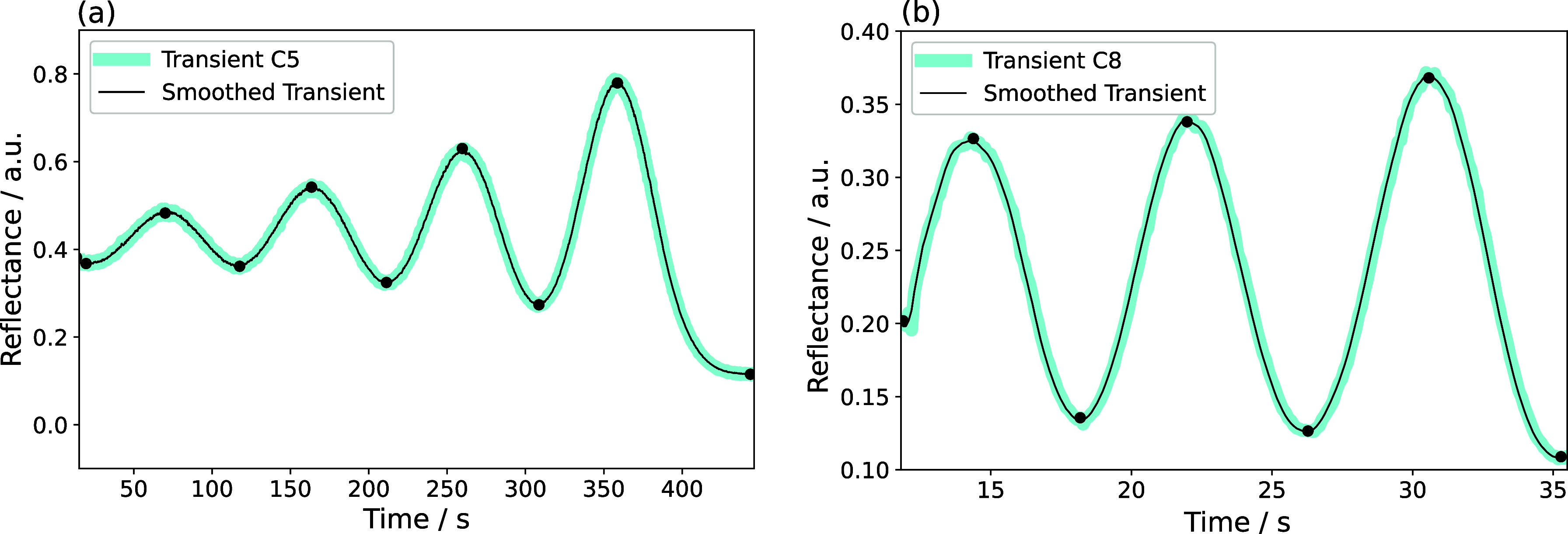
Transients acquired upon etching of GaAsP-based sample C5 at 2.6 eV (volume ratio 1:1:200) and (b) GaAsP-based sample C8 at 2.0 eV (volume ratio 1:1:10). The reflectance transient data are filtered with a Savitzky–Golay filter (black, window length: 14, polynomial: 3). Black dots highlight the extrema.

**Figure 6:**
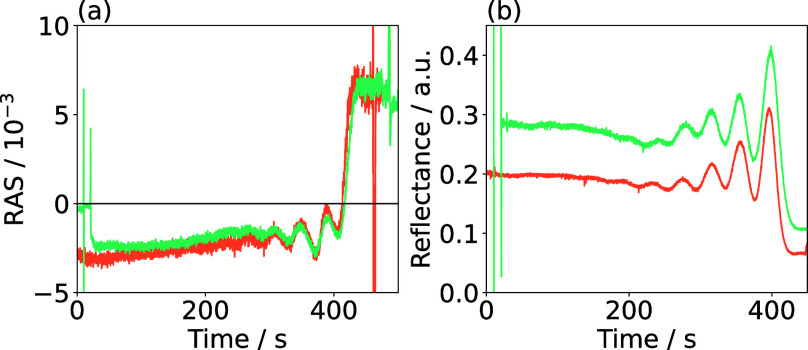
(a) RAS transient during the removal of a GaAs cap layer of two solar cells based on the commercial absorbers from AZUR SPACE at 2.6 eV. (b) Corresponding reflectance transient at 2.6 eV.

## Results and Discussion

### Characterization of Solar Cells before and after Wet Etching

Encouraged by the work of Sombrio et al.,^13,15^ who have used RAS process control during dry etching, we aim to transfer the method to wet-chemical preparation. This is challenging due to the more complex environment created by introducing an additional gas/liquid/solid interface compared to dry etching and the broad parameter space introduced thereby. The oscillating behavior of the reflectance should nevertheless be observable, also for other material systems, in case of a homogeneous etching by correctly chosen experimental conditions. In the following, GaAsP/Si-based multijunction solar cells, which are to be used as photocathodes, are investigated. For the preparation of a photocathode from the provided solar cell, the first step is the wet-chemical etching of the 250 nm thick, highly *n*-doped GaAs_0.8_P_0.2_ cap layer, as depicted in Figure 1. This is also used in photovoltaic processing, where the highly doped cap layer creates both an ideal ohmic contact and a metal diffusion barrier for the contact fingers. Additionally, the cap layer protects the solar cell from mechanical and chemical stresses. Etching away the cap layer exposes the 25 nm thick, *n*-doped Al_0.6_In_0.4_P window layer, which forms a charge-selective contact between the subjacent layer stack and the electrolyte or catalyst. The pristine cap layer surface is investigated prior to the etching process in order to assess the impact of the initial morphology and composition on the etching procedure. For this investigation, RAS, XPS, and AFM were applied as complementary methods. Before etching, the AFM image of the surface shows structural inhomogeneities and a roughness (rms) of 3.2 nm as can be inferred from Figure 2c. After etching, the surface becomes smoother (rms = 0.6 nm, see Figure 2d). Deeper lying inhomogeneities in the layer stack originate in the complexity of the lattice-mismatched growth of GaAsP/Si tandem absorbers via MOVPE. Uncontrolled thermal and lattice mismatch in III–V/Si tandem absorbers typically cause extensive defects in the material.^21^ The lattice mismatch between the GaAsP absorber and the Si substrate is about 3%. This is overcome by means of a step-graded metamorphic buffer. This way, the in-plane lattice constant is increased in a controlled way, thereby reducing the final threading dislocation density in the GaAsP absorber.

The GaAsP/Si-based multijunction solar cells used in the following present a higher-than-optimal threading dislocation density of around 1–2 × 10^7^ cm^–2^. The defect-rich solar cells are, however, well-suited for the assessment of suitable etching procedures, as they react sensitively toward a variation of etching conditions. RAS fingerprint spectra can be used to quantitatively assess the quality of the initial and the etched surface in comparison with spectra from literature and recorded during the growth process (see Figure 2a). The RA spectrum of the pristine sample denoted as C0 in Figure 2a matches well with a highly doped (>10^18^ cm^–3^), oxidized GaAs(001) surface of 0.2° off-cut.^22,23^ Yet, the features at the *E*_1_′ and *E*_1_′ + Δ*E*_1_′ critical points at 2.9–3.2 eV, which are characteristic for the reconstructed or oxidized GaAsP surface, are less pronounced. More prominent are the spectral signatures of the *E*_0_′ and *E*_0_′ + Δ*E*_0_′ transitions at 4.6 and 4.9 eV, related to near-surface-bulk ordering.^23,24^ This indicates an isotropic and amorphous layer on top of the GaAsP cap layer. Therefore, the optical properties of the GaAsP near surface prevail, which leads to a less distinctive peak pattern in the lower energy range (2.9–3.2 eV), while the surface-modified bulk transitions at higher energies (4.6–4.9 eV) are clearly evident. Additionally, the buried heterointerfaces in the provided solar cell can contribute to the anisotropy signal.

The presence of Ga_2_O_3_ and partially As_2_O_3_ on sample C0 detected via XPS (Figure 2b) confirms the formation of a surface oxide layer. This is to be expected as a consequence of the sample handling under noninert conditions.

### Fabry–Pérot Oscillations during Wet Etching

Alternatively to the oxide layer, the low signal-to-noise ratio below 3 eV can be explained by interference phenomena folded on the RA spectrum.^25^ In fact, the heterojunction structure meets the conditions (layer thickness, refractive indices, band gaps) to act as a Fabry–Pérot resonator.^26^ This was already demonstrated for the case of dry etching, showing that the continuous decrease of light path difference between light reflected at the topmost and at the underlying layers leads to an alternation of destructive and constructive interference.^15,27^ Consequently, a smooth wet-chemical etching process and the gradual reduction of the GaAsP layer thickness over time can also be expected to produce constructive and destructive interference, resulting in intensity oscillations. Frequency and intensity are hereby determined by the underlying layer stack and its optical properties as well as the photon wavelength. These oscillations are visible in the “DC signal” as well as in the RA single-energy transients as depicted in Figures 3 and 4, respectively. In this context, the “DC signal” represents a measure for the reflectance (see Supporting Information eq S1), while the RA spectra offer surface structural information. In the following, the reflectance signal is used for the determination of the etching time, depth, and rate. To describe the Fabry–Pérot oscillations, the full multilayer thin film stack and the reflection amplitude at each interface need to be considered. For the first interface of a thin film layer stack, i.e., between the substrate and film, the reflection amplitude can be described as^28^

2 $$r_{2}=\frac{r_{12}+r_{01}\exp\left(-2i\delta_{1}\right)}{1+r_{12}r_{01}\;\exp\left(-2i\delta_{1}\right)}$$

Here, $r_{ij}=\frac{{ñ}_{j}-{ñ}_{i}}{{ñ}_{j}+{ñ}_{i}}$ with ${ñ}_{i}$ the complex refractive index of, and δ_1_ the optical path in, the respective layer. For two layers on a substrate, the same line of thought leads to the following equation:^28^

3 $$r_{3}=\frac{r_{23}+r_{2}\;\exp\left(-2i\delta_{2}\right)}{1+r_{23}r_{2}\;\exp\left(-2i\delta_{2}\right)}$$

The same approach can then subsequently be applied to each additional interface, yielding the following reflection amplitude for a system with *n* layers on a substrate:

4 $$r_{n}=\frac{r_{n-1,n}+r_{n-1}\;\exp\left(-2i\delta_{n-1}\right)}{1+r_{n-1,n}r_{n-1}\;\exp\left(-2i\delta_{n-1}\right)}$$

The reflection coefficient corresponds to the square of the reflection amplitude and reads as

5 $$R=\frac{r_{n-1,n}^{2}+r_{n-1}^{2}+2r_{n-1,n}r_{n-1}\;\cos\left(2\delta_{n-1}\right)}{1+{\left(r_{n-1,n}r_{n-1}\right)}^{2}+2r_{n-1,n}r_{n-1}\;\cos\left(2\delta_{n-1}\right)}$$

Under the assumption of a well-defined etching process, the thickness of the topmost layer is continuously decreasing such that an alternation of constructive and destructive interference is expected. This interference causes Fabry–Pérot-type oscillations of the reflectivity as a function of the etching time and hence the layer thickness (see eq 5). The extrema of the oscillations are determined by the optical paths δ_*i*_, which depend on the photon wavelength λ, as well as the thickness *d*_*i*_ and refractive index *n*_*i*_ of the corresponding (GaAsP) layer as denoted below:

6 $$\delta_{i}=\frac{2\pi \times n_{i}d_{i}\cos\left(\theta \right)}{\lambda }$$

Here, θ corresponds to the angle of incidence and is set to zero (near-normal incidence). This results in the following condition for constructive interference:

7 $$\delta_{i}=\pi k,k\in Z$$

This alternation translates to oscillations of the time-resolved reflectance which result, for anisotropic surfaces, also in oscillations of the RA spectrum. Figure 3a shows a reflectance color plot (CP) acquired between 2.0 and 2.6 eV upon etching of sample C1 (see Table 1) in a solution of H_2_O_2_/NH_4_OH/H_2_O of 1:1:200 volume ratio. This energy range was chosen, since the most pronounced oscillation modulation is obtained here. In Figure 3b, reflectance transients at 2.0 and 2.6 eV, which were extracted from the CP, are depicted. For the two chosen photon energies, the evolution of the oscillation amplitude differs visibly. Over time, the oscillation amplitude only slightly increases in the transient recorded at 2.0 eV, whereas at 2.6 eV, it shows a significant increase in oscillation amplitude by a factor of about 4 (Figure 3b). The latter is assigned to a decrease in light absorption by the shrinking photoabsorber thickness upon etching and is well reproduced by eq 5 when reflection at three different interfaces (GaAsP/AlInP, AlInP/GaAsP, GaAsP/water) is assumed. Closer to the band gap energy of GaAs_0.8_P_0.2_ (1.6 eV), this phenomenon is less pronounced because of the lower absorption coefficient (see Supporting Information Figure S2).^29^ Therefore, the reflectance signal at 2.0 eV is only slightly affected by the decreasing layer thickness of the GaAs_0.8_P_0.2_ cap layer as compared to the signal observed at 2.6 eV.

**Table 1:** Etching Parameters of the Discussed GaAsP-based Solar Cells in H_2_O_2_/NH_4_OH/H_2_O Etching Solution

sample	C1	C2	C3	C4	C5	C6	C7	C8
volume ratio	1:1:200	1:1:200	1:1:200	1:100:10,000	1:1:200	1:1:200	1:1:10	1:1:10
contact time (s) ± 5 s	550	1600	400	5648	600	505	260	55

### Determination of Etching Time, Depth, and Rate Using Reflectance

In agreement with the case of MOVPE growth and reactive ion etching procedures,^15,24^ well-defined Fabry–Pérot oscillations are observed during wet-chemical etching, as can be inferred from the reflectance and RAS CP and transient measurements depicted in Figures 3 and 4a,b, respectively. This demonstrates a well-defined etching behavior and emphasizes the ability for process control in a wet-chemical environment using reflectance and RAS measurements. The number and period of the observed oscillations are wavelength-dependent, which allows us to precisely determine the etching parameters—the etching time, depth, and rate.^15^ Furthermore, the RAS amplitude does, in principle, allow the continuous monitoring of the preservation of structural order and hence etching homogeneity.^20^

The time needed to remove the GaAsP cap layer, in the following referred to as “etching time”, is in general shorter than the actual “contact time” between the photoabsorber and etching solution, because during the experiment, a certain time passes for the etching solution removal and washing steps. Ideally, selective etching solutions are applied, with a much higher etching rate for the dissolved layer than for the subjacent one and, which only provide a small difference between the anisotropic etch rate along defects and isotropic layer-by-layer etching, to account for this fact.^30^ Wet-chemical etching with in situ reflectance and RAS control can be applied in two different scenarios:

1. In the case of a known cap layer thickness *d* and known material to be removed with the refractive index *n*(λ), the expected number of oscillations *k* at a defined photon energy λ can be calculated via eq 8 as

8 $$k=\frac{d\times 2n\left(\lambda \right)}{\lambda }$$

The measurement is stopped when the calculated number of oscillations is completed. In addition, the oscillation period τ can be determined from the first observed oscillation and used to calculate the expected etching time, *t*_etching_.

9 $$t_{etching}=k\times \tau$$

2. In case of an unknown layer thickness *d* and a known material to be removed, etching is stopped when the oscillation of the reflectance transient finishes. The start- and end point of the oscillations are subsequently determined from the reflectance transients’ rate of change as described in the Supporting Information Section S3.2.1. This then also allows us to extract the etching time, *t*_etching_. From the extrema of the reflectance transient, the number of full oscillations, *m*, and the average duration of one oscillation, τ, are determined. The total number of oscillations, *k*, is evaluated as follows:

10 $$k=m+\frac{t_{etching}-\left(m\times \tau \right)}{\tau }=\frac{t_{etching}}{\tau }$$

Knowing the total number of oscillations, the etching depth can be determined in hindsight:

11 $$d=\frac{\lambda \times k}{2n\left(\lambda \right)}$$

In the following, this procedure will be applied for the photocathode preparation with the removal of a GaAsP cap layer. The cap layer thickness is determined experimentally, as described in case 2 above. Time-resolved reflectance measurements are performed at 2.0 and 2.6 eV upon etching of samples C5–C8 as depicted in Figure 5a,b, allowing for the determination of the etching parameters. After determining the etching time and the number of oscillations, the etching depth and ratio are calculated, following the procedure described in Supporting Information Section S3.2.1.

Table 2 resumes the etching parameters determined by analyzing transients measured at two different photon energies for two different etching ratios, including the ones presented in Figures 5 and 6. The GaAsP cap layer thickness, estimated from the growth conditions, amounts to 250 ± 12 nm for samples C5–8 and 500 nm ± 25 nm for samples A1–4. For the samples C5–8, the transient analysis at 2.6 eV yields etch-depths of 255.5 and 245 nm for the solutions with 1:1:10 and 1:1:200 etching ratios, while etch-depths of 238.1 and 241.3 nm are obtained from the 2 eV transients for 1:1:10 and 1:1:200 etching ratios. This results in an overall standard deviation of 7.6 nm for the etching depth for samples C5–8. It is noteworthy that the increased etching rate - by a factor of 15.7 using a 20 times higher concentrated etching solution - does not significantly reduce the precision in determination of the etching depth. The etching rates determined for the two different etching solutions are deduced from two independently measured transients at 2 and 2.6 eV, leading to very similar values. The etching rate in the 1:1:200 etching solution is determined to be 0.63 ± 0.04 nm/s, whereas a rate of 9.9 ± 0.04 nm/s is found for the 1:1:10 etching solution. Therefore, the whole concentration range can be considered appropriate for precise etching. For the subsequently etched GaAs-based samples A1–4 prepared under the same conditions (1:1:200), the etching rate is 1.18 ± 0.08 nm/s and the determined mean etch depth is 509 ± 8.3 nm.

**Table 2:** Etching Parameters of Samples C5–C8 and A1–4 Determined Using the First and Last Extremum of the Oscillation as Respective Start and End Points^a^

H_2_O_2_/NH_4_OH/H_2_O	1:1:200		1:1:10			1:1:200		
sample N°	C5	C6	C7	C8	A1	A2	A3	A4
photon energy (eV)	2.6	2.0	2.6	2.0		2.6		
etch-time/s	430	357.4	25.8	23.4	421.6	411.4	457.1	446.8
etch-depth/nm	255.5	238.1	245	241.3	524	517.5	486.3	511.8
error (nm)	3.2	3.4	3	3.4	6.2	6.1	5.7	6
etch-rate/(nm/s)	0.6	0.7	9.5	10.3	1.2	1.3	1.1	1.1

a The etching rate is calculated then by averaging over the last 2.5 oscillations.

The given precision allows for an in situ etch-depth determination and is surprisingly even higher than the first reported precision of 200 ± 16 nm for dry etching processes using a comparable method. This is suitable for process control and constitutes a powerful tool to identify sample-to-sample variations, e.g., concerning layer thicknesses within one wafer or varying etch rates due to crystal defects or adsorbates. To even increase the precision of the etch depth determination statistically, several transients at different wavelengths can be analyzed.^15^

### Optimizing Etching Procedures Using RAS In Situ

In order to establish a reproducible etching routine for a given material system, the physical and chemical parameters need to be optimized toward a homogeneous material removal. Therefore, large selectivity of the etching solution toward the material to be etched and slow etching rates along defects should be aimed for. For quality assessment of the etching procedure, concise postanalysis is performed. In the following, we show that in situ RAS control can supply direct feedback for the optimization of etching procedures. For this purpose, etching solutions with three different H_2_O_2_/NH_4_OH/H_2_O ratios and adapted contact times were investigated.

#### Volume Ratio 1:1:10

For sample C8, the most highly concentrated etching solution with a 1:1:10 volume ratio and a contact time of 55 s is investigated. Etching was stopped when the AlInP window layer was reached using RAS in situ control. This is confirmed by In and Al peaks in the XPS spectra (see Figure 2b) and Supporting Information Figure S5. The corresponding RA spectrum in Figure 2a shows a broad peak between 3.2 and 4.8 eV which can be attributed to an overlap of features stemming from surface-modified bulk electronic states. Surface transitions are possibly preventing the identification of bulk critical point transitions of AlInP at 3.8 eV and 4.8 eV.^31^ At 4.3 eV, where a GaAsP feature is expected, the RA spectrum of C8 shows a shoulder. Therefore, the cap layer is either not fully removed and the residuals cause that feature or the subjacent absorber by a contribution of the buried window/absorber interface must be considered. The shoulder is not observed for the RA spectrum recorded during the growth of the solar cell in the MOVPE chamber (see Figure 2a “In Growth”), indicating that the window/absorber interface can be considered not detectable via RAS under the given conditions and therefore cannot be the origin of the feature. Additionally, Ga and As oxides are identified via XPS (see Figure 2b) at the very surface, indicating an incomplete cap layer removal. This is in line with a possible surface-modified bulk transition of Ga oxides, which have formed on the surface, corresponding to the shoulder feature at around 4.5 eV.^32,33^ Referring to optical microscope images, it is to be suspected that there are three morphological structures identifiable on the surface: cap layer residuals on top, the window layer, and etch pits exhibiting the subjacent GaAsP absorber (see Supporting Information Figure S6).

#### Volume Ratio 1:1:200

Consequently, the concentration of the etching solution is reduced alongside a prolonged contact time to increase the homogeneity of the process and diminish etch pits. Again, successful removal of the GaAsP cap layer, revealing the AlInP window layer, is clearly reproduced by comparing the RA spectra of solar cells C0 and C1 before and after etching (H_2_O_2_/NH_4_OH/H_2_O, volume ratio 1:1:200), as depicted in Figure 2a. Accordingly, sample C1 exhibits the peak pattern of AlInP with the main feature at 3.6 eV, in contrast to the main feature of GaAsP at 4.3 eV. Features in this energy range are attributed to the near-surface characteristics. The generally low anisotropy below 2.8 eV (partly) originates from the presence of Ga and As oxide residuals at the surface (see Supporting Information Figure S5). Nevertheless, after etching, a topographically smooth surface is achieved (rms = 0.6 nm) compared to the initially relatively rough GaAsP surface (rms = 3.2 nm), as depicted in Figure 2c,d.

Comparison to an RA spectrum measured during the final steps of the AlInP layer growth (see Figure 2a “In Growth”) substantiates the analysis of the spectra after etching. It can be confirmed that the AlInP window layer is reached and shows a similar surface structure for sample C1. The minima of the RA spectra after wet-chemical etching in water are blue-shifted at 2.8–3 eV compared to the spectrum recorded ex situ under a N_2_ atmosphere, interrupting the growth of the solar cell. These differences are not surprising, as a certain impact of the etching solution on the AlInP surface reconstruction is to be expected.^19^

#### Volume Ratio 1:100:10,000

For etching with an even more dilute solution of a changed volume ratio 1:100:10,000 (C4), no change in the RA spectrum can be observed over a period of 1.5 h as depicted in the color plot in Supporting Information Figure S7. Importantly, there is no oscillating behavior visible from the RAS CP, which confirms that no well-ordered etching is taking place. This supports the hypothesis that changing the volume ratio of the etching solution has an influence on the surface chemistry of the etching process and subsequently the surface morphology, not just the etching rate. However, this is in contrast to the XPS results presented in Figure 2b, which show a loss of GaAs and As_2_O_3_ features in favor of arising InP, GaP, In, and Al peaks for sample C4.

The etching solution is diluted here by a factor of 50 compared to the 1:1:200 case and a factor of 1000 compared to the 1:1:10 case with respect to the H_2_O_2_ content. Since H_2_O_2_ oxidizes the surface, while NH_3_ subsequently complexes the ions to dissolve the layer, one can assume that the oxidation of the surface is slowed down with maintaining the ability for solvation of surface species. The choice of the etching volume ratio is after all relevant for creating conditions that support the formation of an equilibrium between oxidation and dissolution, resulting in a homogeneous topography. Additionally, the duration of contact significantly impacts the structural evolution during etching and hence surface anisotropy and roughness. From an SEM cross-section (see Supporting Information Figure S8), it is visible that the GaAsP cap layer is still present in the investigated profile. Therefore, the detection of Al and In on the sample surface via XPS can be attributed to pinholes set forth in the very low-concentration etching solution, revealing the AlInP layer. In addition to the thereby disclosed AlInP areas, dissolved Al and In species can be redeposited on top of the GaAsP layer. This finding is backed up by sample C9, which was etched under the same conditions for half of the time (47 min). Likewise, the RA color plot does not show any oscillation but stays constant over the course of the measurement. Referring to the XPS results of C9, the GaAsP peaks are still dominant with no Al or In features detected (see Supporting Information Figure S9).^34,35^ The AFM images in Supporting Information Figure S10 show an increase in surface roughness by a factor of about 2 for C4, compared to the starting surface depicted in Figure 2d. Multiple spike-like structures occur. Considering a lower oxidation speed for C4, a more inhomogeneous etching can be explained by a self-promoting increase of the surface topology under the given conditions. Existing defects quickly expose subjacent layers, which provide differing etching rates to the etching solution. The GaAsP absorber below the AlInP window layer has a significantly higher etching rate in the given etching solution. Hence, in the considered case of GaAsP wet etching with a H_2_O_2_/NH_4_OH/H_2_O 1:100:10,000 solution, reducing the H_2_O_2_ content decreases the homogeneity of the process, increases roughness, and shifts the etching from anisotropic to isotropic.

#### Additional Washing Step for Oxide-Free Surfaces

As mentioned before, the initial GaAsP cap layer surface shows Ga oxides (see Figure 2d), formed due to exposure to atmospheric oxygen and water vapor. The solar cells were exposed to air before wet-chemical etching and were afterward transported under a N_2_ atmosphere, which does, however, not allow for a fully inert transfer to the XPS chamber. Still, it was not expected that also all etched samples investigated by XPS exhibit Ga_2_O_3_ and As_2_O_3_ residuals on the window layer surface (see Figure 2d). One hypothesis for their origin is redeposition from the etching solution. This is supported by the fact that even on samples, which were in contact with the etching solution much longer than necessary for the complete removal of the GaAsP layer, e.g., sample C2 (see Figure 2b and Table 1), traces of Ga_2_O_3_ and As_2_O_3_ are found. During the etching procedure, the GaAsP surfaces are oxidized by H_2_O_2_, and the oxides are subsequently solvated by ammonium ions.^30,36^ To remove residual oxides, an additional washing step with an aqueous ammonia solution (3.3 mol/L, contact time 60 s) was introduced. Working effectively, this was established as the last step of the etching routine to avoid oxide residuals. This is showcased on the GaAs-based samples A1 and A2 , with a 500 nm GaAs cap layer and a 25 nm AlInP window layer (see etching observation in RAS transients Figure 6a and DC transients Figure 6b, XPS evaluation in Supporting Information Figure S11). The washing step was not yet implemented for all of the other experiments described here.

### Deviations from Oscillating Behavior Due to Initial Structural Constraints

Samples C1 and C2 are prepared under the same etching conditions (volume ratio 1:1:200). Despite this fact, the RA and reflectance transients recorded during the etching of C1 and C2 at 2.6 eV differ considerably, as depicted in Figures 3a, 5b and 7a,b, respectively. In contrast to Figure 3, the anisotropy and non-normalized reflectance measured during the etching of C2 are not oscillating with the expected frequency. Instead, a single, high amplitude “oscillation” is observed in the transient at 2.6 eV, while the reflectance continuously drops until it reaches a plateau at nearly zero, followed by a slow increase toward the end of the cap layer etching. This points out that the characteristics of anisotropy and reflectance oscillations are also dependent on the initial sample surface quality. Sample C3 showed a comparable behavior to C2 under the same etching conditions. In order to understand the occurrence of the high RAS values in the transient of C2 and to study the influence of the morphology during etching, for sample C3 the procedure was interrupted after the anisotropy reached its maximum value to investigate the surface preserved in this stage.

**Figure 7:**
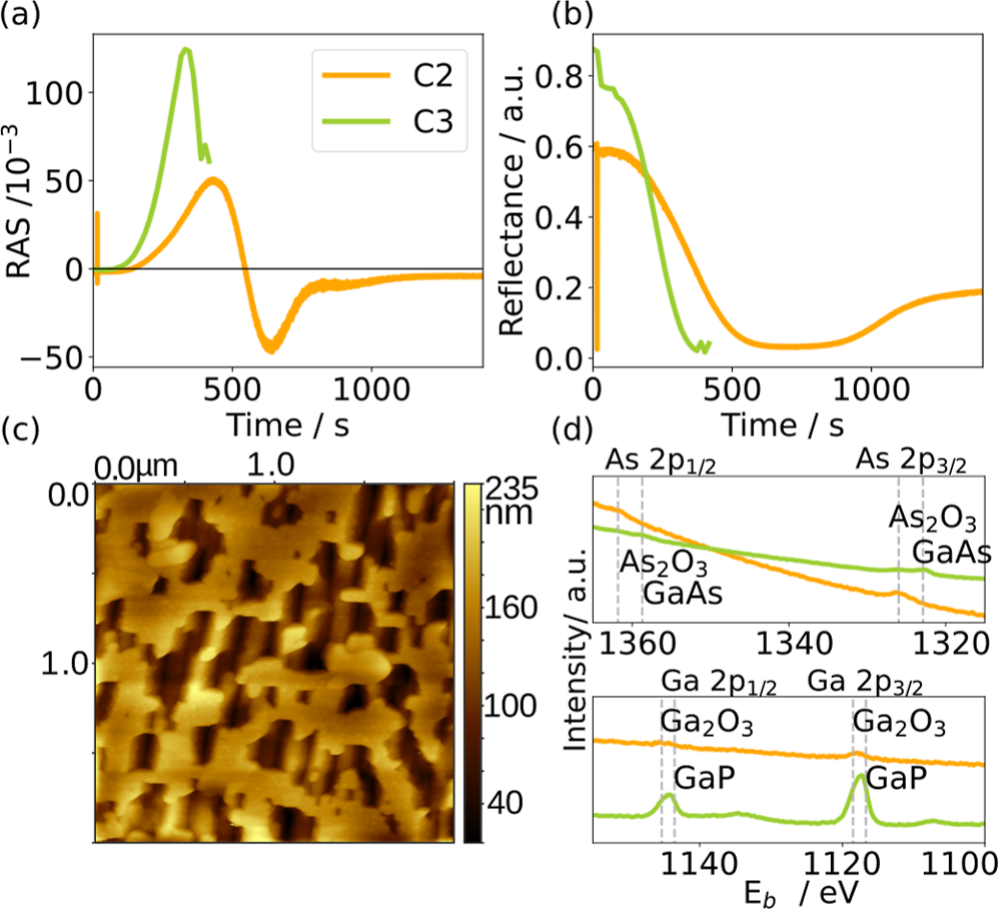
(a) RAS transients and (b) reflectance signals of GaAsP-based samples C2 (fully etched) and C3 (etching stopped in the cap layer) taken at 2.6 eV and for an etching ratio of 1:1:200. (c) AFM image of GaAsP-based sample C3 after the etching procedure was stopped. (d) XPS analysis of In Auger, As 2p, and Ga 2p core levels for C2 and C3, supporting that the etching was stopped within the GaAsP cap layer for C3.

The transient of C3 qualitatively resembles the one of C2, but its RAS amplitude is even 2.5 times higher. Large increases in RAS amplitude with simultaneous drops in reflectance have been attributed to increasing surface roughness in literature.^17^ Comparing the AFM images of C3 in Figure 7c and C1 in Figure 2d, the surface of C3 is found to be three to four times rougher than that of C1 (see Table 3). A roughening of the surface can translate to an increasing RAS signal if it is anisotropic. This can also be seen from the McIntyre–Aspnes relation which reads as follows:^38^

Re⁡(Δrr)=4πdλIm[εs,x−εs,y1−εb] 12

with ε_*s*,*x*_ – ε_*s*,*y*_ describing the dielectric anisotropy of the surface, ε_*b*_ the dielectric function of the bulk material, and *d* the thickness of the anisotropic surface layer.

**Table 3:** Root Mean Square Roughness of Homogeneously (C1) and Inhomogeneously (C3) Etched Samples Compared to Initial Surface C0

sample N°	C0	C1	C3
RMS roughness (Sq)/nm	3.2	0.6	40.5

Along the etching direction, two differently patterned layers are found for C3. These patterns indicate a locally faster vertical etching, causing a partially preserved ordering of the surface. Therefore, an increasing rms roughness correlates here with an anisotropic etching. This causes the growing thickness of the anisotropic surface layer, *d*, which is directly proportional to $\frac{\Delta r}{r}$ (see eq 12).^17^ Here, the strong RAS feature is not wavelength-specific and can be observed at multiple energies (see Supporting Information Figure S12). This substantiates the surface roughening, rather than a specific sensitivity to Ga dimer or As dimer accumulation, as it was observed in previous studies.^37^ Furthermore, the XPS spectra of C3 (see Figure 7d) do not support an As-rich surface, opposing the observation of As dimer accumulation at 2.6 eV. The anisotropic etching is suspected to be caused by a sample initially rich in defects, which is in line with the fact that the investigated samples exhibit a significant amount of pinholes (see Figure 2d). This is therefore also the reason for the different behavior of C1 compared to C2 and C3, despite the same procedure being applied.

## Conclusions

In this work, we have shown for the first time that Fabry–Pérot oscillations of reflectance and RAS transients can be observed in a wet-chemical environment. The observed oscillations are sharply featured and reproducible, with the reflectance oscillations offering a better time resolution and signal-to-noise ratio than the RAS oscillations.

Therefore, we used the reflectance oscillations to determine etching depth, time, and rate in situ. The depth can hereby be determined with a precision of ±8.3 nm or less and an error of 6.2 nm or less for the described removal of epitaxial III–V layers from photoelectrode surfaces. The accuracy could thereby be enhanced by evaluating several transients recorded in parallel. In the exemplary case of GaAsP wet etching with a H_2_O_2_/NH_4_OH/H_2_O solution, a concentration of 1:1:10 creates too harsh conditions. The surface is roughened during etching, and the window layer is damaged. Lowering the concentration of the etching solution to 1:1:200 keeps the equilibrium of oxidation and dissolution stable at a slower etching rate, resulting in a smooth topography and well-controllable etching times. At even lower concentrations of 1:100:10,000, the etching is extremely slow, not resulting in the removal of the cap layer in the investigated time frame but in a surface roughening. The changed ratio between H_2_O_2_/NH_4_OH leads to a lower ability for oxidation compared with dissolution. This is in line with the observed redeposition of dissolved metals (In, Al) on the cap layer surface. Consequently, the formation of etch pits is suspected.

Imbalancing the etching process by changing the ratio between H_2_O_2_/NH_4_OH and the etching rate decreased the homogeneity of the resulting surface. This shift is observed by a loss of the defined oscillation pattern. Therefore, reflectance Fabry–Pérot oscillations are a tool for etching condition optimization toward homogeneous etching. The observation of RAS and reflectance oscillations under adequate etching conditions were reproduced for a different layer stack of GaAs/AlInP photoabsorbers, demonstrating the robustness of the method toward more complex multilayer stacks.

In situ RAS is furthermore used to investigate sample quality, sample-to-sample variations, and surface roughening. Deviations from the expected oscillation pattern indicate an inhomogeneous etching process, e.g., a roughening of the surface or a loss of anisotropy.

The presented method offers an in situ, easy-to-apply, noninvasive access to process control and reproducibility assessment in a wet-chemical environment. The real-time evaluation of etching quality with reflectance and RAS upon wet etching is a reliable, systematic, and simple tool for the functionalization of photoelectrodes. Requirements for investigated material systems are a homogeneous etching and a well-defined interface between bulk and the material to be removed, such as a solar cell grown on a well-defined substrate. Future studies could extend the method to other monocrystalline material systems together with a low-absorbing etching solution to assess its applicability in other domains of semiconductor fabrication. Interesting examples of such possible fields of application are the production of white and blue LEDs, where an anisotropic wet-chemical etching increases the device performance,^5^ the manufacturing of microelectromechanical systems (MEMS), and III–V semiconductor-based optoelectronics.^6^ As soon as the general processing and involved optical signatures for a given system are established, it is then in principle possible to simplify the setup and simultaneously increase time resolution by the use of single-wavelength light sources such as laser diodes.

## Supplementary Material



## Notes

Additional experimental details, materials, and methods, including photographs of experimental setup, (scanning electron) microscope pictures, and additional RAS spectra; ref 8 is cited in the Supporting Information. Data for this article, including spectroscopic data (RAS, XPS) and AFM data, are available on Zenodo at [10.5281/zenodo.21276051]. (PDF)

## ⊥

NexWafe GmbH, Hans-Bunte-Strasse 19, 79108 Freiburg im Breisgau, Germany
