# Supplementary material for: In Situ Process Control during Wet-Chemical Etching of Heteroepitaxial III–V Layers Using Reflection Anisotropy Spectroscopy
Source: J Phys Chem A. 2026 Jul 31;130(32):6465–74. doi: 10.1021/acs.jpca.6c02573 (PMC13479611; doi:10.1021/acs.jpca.6c02573)
Supplement: Supplementary file 1 [file jp6c02573_si_001.pdf]

In Situ Process Control during Wet-Chemical Etching of Heteroepitaxial III-V Layers using Reflection Anisotropy Spectroscopy

Erica A. Schmitt,[1], ‡, * Margot Guidat,[1],†,‡ Marco Flieg,[1] Jonas Grutke, [3] Maximilian Diecke,[1] Kristof Möller, [4] Holger Euchner,[1] Matthias M. May*[1,2]

*Email: erica-anne.schmitt@uni-tuebingen.de
Phone: +49 7071 29-76332

[1] Institute of Physical and Theoretical Chemistry, Universität Tübingen, Auf der Morgenstelle 15, 72076 Tübingen, Germany.

[2] Center for Light-Matter Interaction, Sensors and Analytics LISA+, Universität Tübingen, Auf der Morgenstelle 15, 72076 Tübingen, Germany

[3] Fraunhofer Institute for Solar Energy Systems ISE, Heidenhofstr. 2, 79110 Freiburg im Breisgau, Germany.

[4] AZUR SPACE Solar Power GmbH, Theresienstraße 2, 74072, Heilbronn, Germany

[†] Present address: NexWafe GmbH, Hans-Bunte-Strasse 19, 79108 Freiburg im Breisgau, Germany.

[‡] These authors contributed equally to this work.

Table of Contents

1 Introduction **S2**

2 Experimental Methods **S2**

2.1 Reflection Anisotropy Spectroscopy Setup . . . S2

2.2 Sample Preparation . . . S2

2.3 *In-Situ* Etching Control Procedure . . . S3

3 Results and Discussion **S4**

3.1 Fabry-Pérot Oscillations During Wet Etching . . . S4

3.2 Determination of Etching Time, Depth and Rate using Reflectance . . . S4

3.2.1 Evaluation of the Etching Time with Unknown Layer Thickness . . . S4

3.2.2 Evaluation of the Etching Time with known Layer Thickness . . . S7

3.3 Optimizing etching procedures using RAS *in-situ* . . . S8

3.3.1 Volume Ratio 1:1:10 . . . S8

3.3.2 Volume Ratio 1:1:200 . . . S10

3.3.3 Volume Ratio 1:100:10 000 . . . S10

3.3.4 Additional Washing Step for Oxide Free Surface . . . S14

3.4 Deviations from oscillating behaviour due to initial structural constraints . . . S15

3.5 Analytical Methods . . . S16

1 Introduction

2 Experimental Methods

2.1 Reflection Anisotropy Spectroscopy Setup

The RA-spectra were measured with an RA-Spectrometer (EpiRAS from Laytec) equipped with a Xenon light source. The setup used for the *in situ* etching monitoring can be found in a previous publication.[8]

The reflectance $R = |r^2|$ as the real square of the Fresnel reflection amplitude is probed in parallel, using the normalized DC signal

$$R = R_{\text{sample}} = \frac{U_{\text{DC}}}{A_{\text{f}}} \tag{1}$$

The measured DC signal is hereby directly proportional to R. To determine the device constant A_f, a reference spectrum would be needed, which was not measured here.

2.2 Sample Preparation

For samples C1-4, C7 and C8, a PEEK (Poly Ether Ether Keton) machined sample holder with 4 magnets for closing is used. The sample is sealed at the front and back side using a Viton® (Fluoroelastomer) sealing mat and a FFKM (Perfluoroelastomer) sealing ring. The exposed area is 38.4 mm ^{2}. The wire was connected to the scratched backside of the 1x1 cm^2 solar cell with silver resin (Ferro GmbH) and stabilized with Kapton® tape (see SI Figure S1). For samples C5 and C6, a glass rod (outer diameter: 6 mm) was cut to length with a glass rod cutter. A copper cable was skinned on both ends and then inserted into the glass rod. The 1x1 cm^2 solar cell was positioned underneath the glass rod on a PTFE

(Polytetrafluoroethylen) mount. The wire was then connected to the scratched backside of the solar cell with silver resin (Ferro GmbH) and stabilized with Kapton® tape. Afterwards, the glass rod was attached to the absorber with epoxy resin "Epoxies 10-3046" (Epoxies Etc.). The Epoxy was cured for 30 min at ambient conditions. Back side, solar cell edges and skinned wire were fully covered with epoxy and left to dry at ambient conditions over night. Epoxy-covered areas were fully covered again and left to cure for 24 h at ambient conditions.

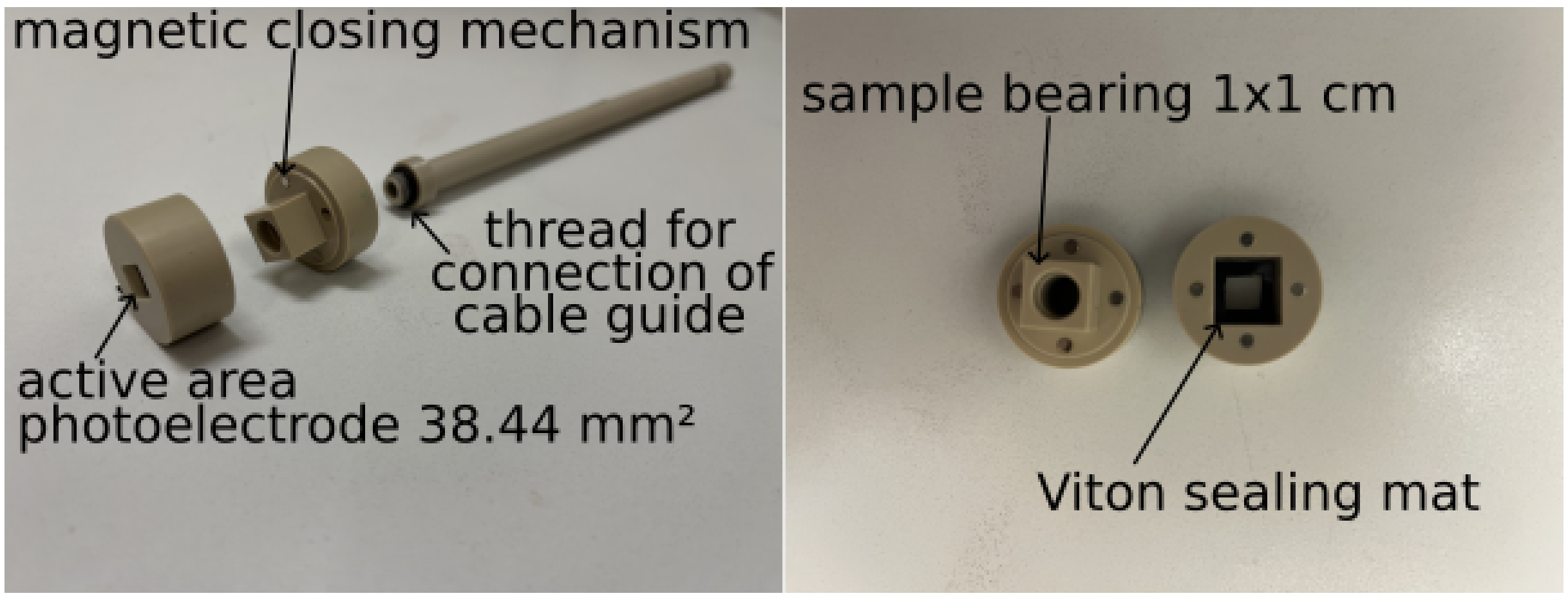

Figure S1: Self-designed PEEK sample holder with magnetic closing mechanism for glue free sample handling.

2.3 *In-Situ* Etching Control Procedure

The reference spectrum of the solar cell corresponding to the GaAs surface was taken in water during the cleaning process of the cell. For the etch monitoring, a solution of 1:1:200 (H_2O_2:NH_4OH:H_2O; H_2O_2 supplied by Merck 30 %, NH_4OH supplied by Merck 25 %) was prepared. Before starting the transient, the PEC cell was filled with the etching solution just below the bottom of the solar cell. 10 s after the start of the transient, the solar cell was fully covered with the etching solution. 30 s after the sample was fully covered, the etching solution was removed. The spectral signature of AlInP can slightly vary depending on the time of contact of the etching solution with the surface of the PEC cell. To avoid significant variation of the AlInP spectrum and to prevent the etching of AlInP by the residual etching solution present on the surface of the solar cell, the PEC cell was rinsed three times with ultra-pure water within 1 minute after the etching process was completed. The spectrum after etching was measured in water during the third cleaning step. Note that for all spectra, a baseline correction is applied to the RA-spectra and to the transient by subtracting the sum of two spectra where the sample has been rotated by 90° in between.

3 Results and Discussion

3.1 Fabry-Pérot Oscillations During Wet Etching

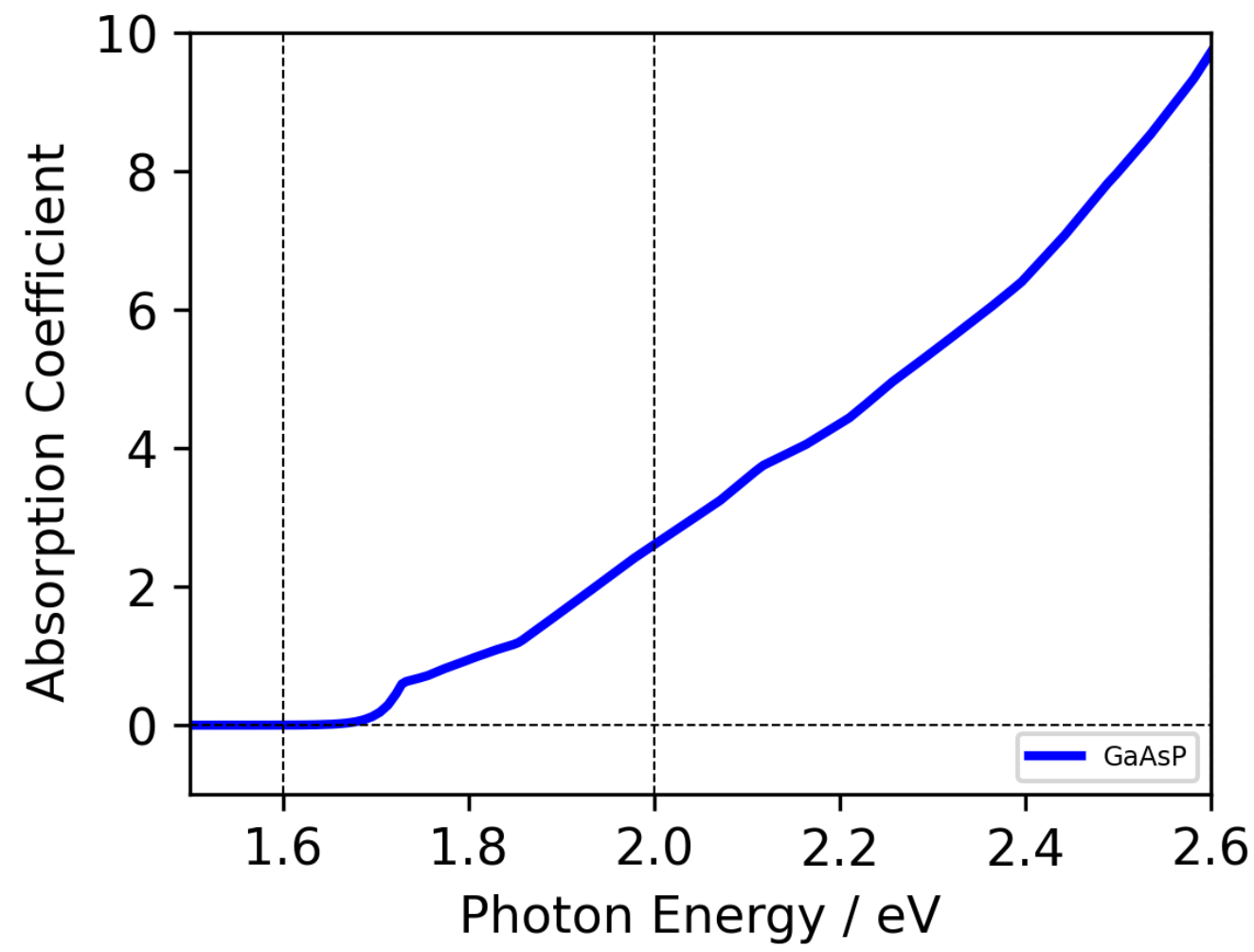

Figure S2: Absorption coefficient over photon energy.

3.2 Determination of Etching Time, Depth and Rate using Reflectance

To reduce the noise of the signal, the DC transient is filtered with a Savitzky-Golay filter (Filter: 10, polynomial: 3)

3.2.1 Evaluation of the Etching Time with Unknown Layer Thickness

- Evaluation of the Etching Time By Using Extrema
  The beginning of the etching time is defined as the first extremum detected during the measurements. This first extremum is caused by the insertion of etching solution to the Schlenk cell which causes a spike in the DC signal due to sudden reflectivity changes. The end of the etching is defined by the end of the last oscillation which shows the transition from GaAsP to AlInP. This point can be determined by using the last extremum detected.

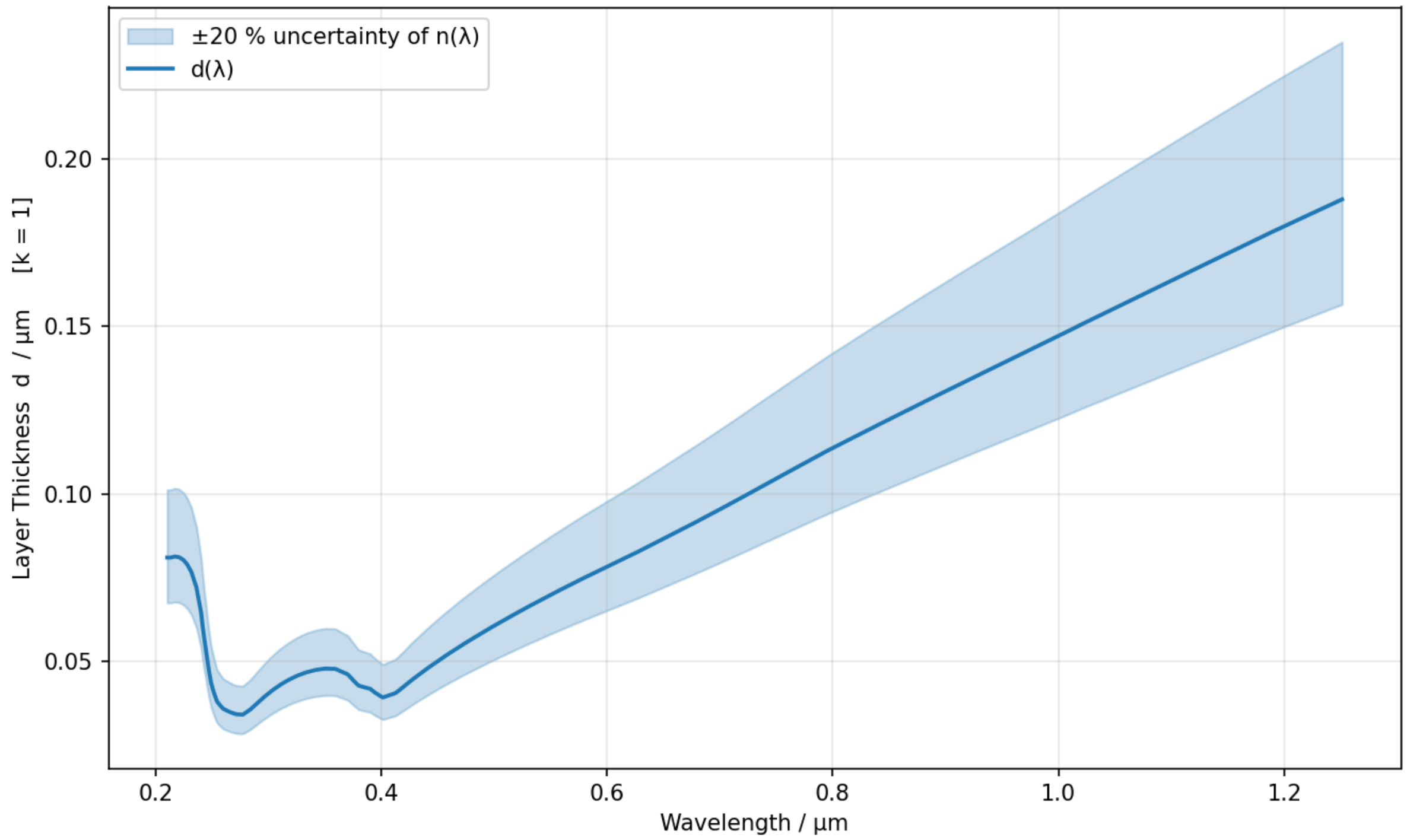

Figure S3: Layer thickness of a GaAsP layer with error bar from uncertainty of n(λ).

- Evaluation of the Etching Depth
  Knowing the etching time, the number of oscillations is determined as followed:
  - The number of extrema is determined and located
  - The number of completed oscillations m is determined
  - Using the extrema, the average wavelength of the oscillation is determined from the last 2.5 oscillations (6 points), starting from the second to last point. For smaller layers, also fewer oscillations could be considered.

  The number of oscillation k is then evaluated as follows:

$$k = (t_{etching} - m * \tau)/\tau \tag{2}$$

  Knowing the measuring wavelength λ, the refractive index n of GaAsP at this wavelength, and the number of oscillations k, the etching depth is calculated with Equation 3.

$$d = \frac{\lambda * k}{2n(\lambda)} \tag{3}$$

Finally, the ratio of the etching depth and the etching time yields the etching rate.

The minimum detectable layer thickness depends on the available probing wavelength and is defined as d(k=1) in equation 3. For samples C5-8, this leads to a minimum layer thickness of d_{min} = 56 nm at 2.6 eV and d_{min} = 81.6 nm at 2 eV. For materials of higher (lower) refractive index, the minimal

thickness will decrease (increase) accordingly.

Statistical evaluation of depth measurements:

- Averaging the measurements C5 -8 yields a mean thickness of 245 nm with a standard deviation of 7.6 nm (k = 1; expanded 95% interval $\pm$ 12 nm), which characterizes the precision of the measurement. Compared with the reference value of 250 nm$\pm$ 12.5 nm (1σ), the result shows a relative deviation of -2%. The mean therefore agrees with the true value within its uncertainty.

- Averaging the measurements A1-4 yields a mean thickness of 509 nm with a standard deviation of 16.5 nm (k = 1; expanded 95% interval $\pm$ 26 nm), which characterizes the precision of the measurement. Compared with the reference value of 500 nm$\pm$ 25 nm (1σ), the result shows a relative deviation of 2%. The mean therefore agrees with the true value within its uncertainty.

3.2.2 Evaluation of the Etching Time with known Layer Thickness

The DC signal drops when etching starts. By that, the starting point of the etching is determined. The expected amount of oscillations is calculated with the known thickness of the layer to be removed. The average duration for one oscillation is calculated over all observed oscillations and by that, the endpoint can be determined.

$$k = \frac{d * 2n}{\lambda} \tag{4}$$

$$t_{etching} = k * \tau \tag{5}$$

This makes it possible to predict the expected number of oscillations and in practice stop the experiment using a well defined parameter.

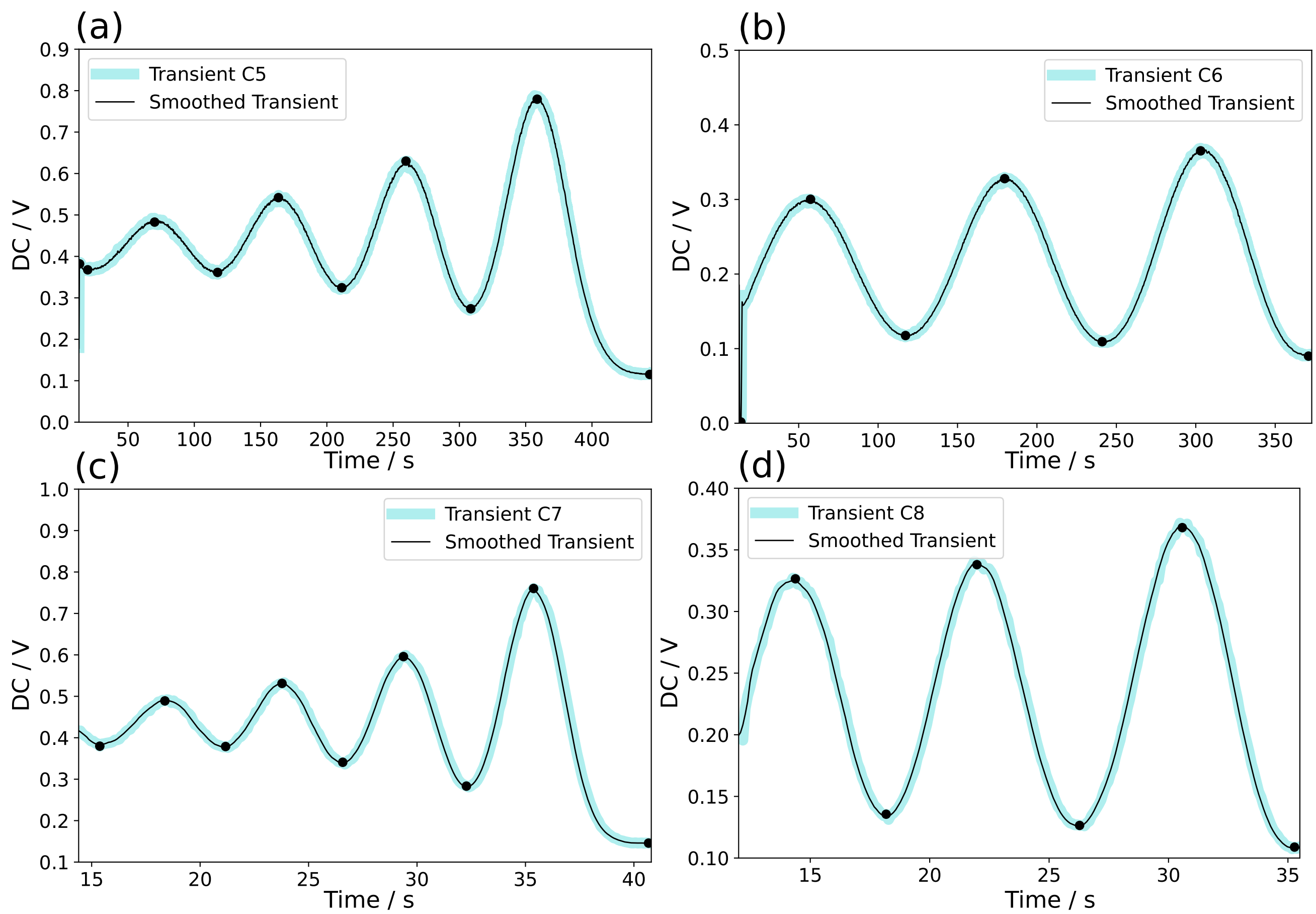

Figure S4: DC transients acquired upon etching of a) C5 at 2.6 eV (etching ratio 1:1:200), b) C6 at 2.0 eV (volume ratio 1:1:200) and c) C7 at 2.6 eV (etching ratio 1:1:10) d) C8 at 2.0 eV (etching ratio 1:1:10). The black curves correspond to the transient data filtered with a Savitzky-Golay filter (Filter: 10, polynomial: 3). The black dots localize the extrema of the oscillations.

3.3 Optimizing etching procedures using RAS *in-situ*

3.3.1 Volume Ratio 1:1:10

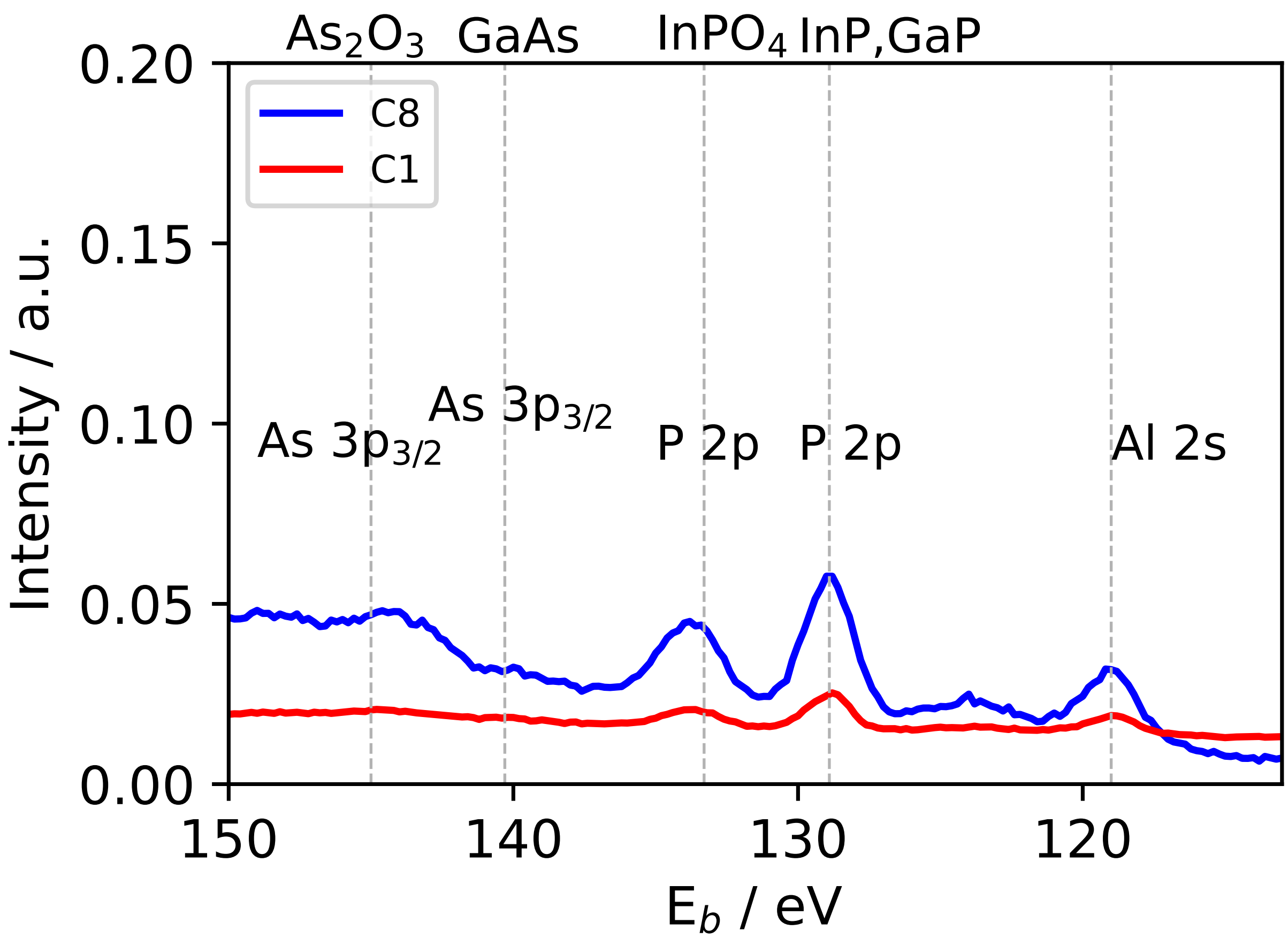

Figure S5: XPS spectra of C1 (1:1:200/540s) and C8 (1:1:10/30s).

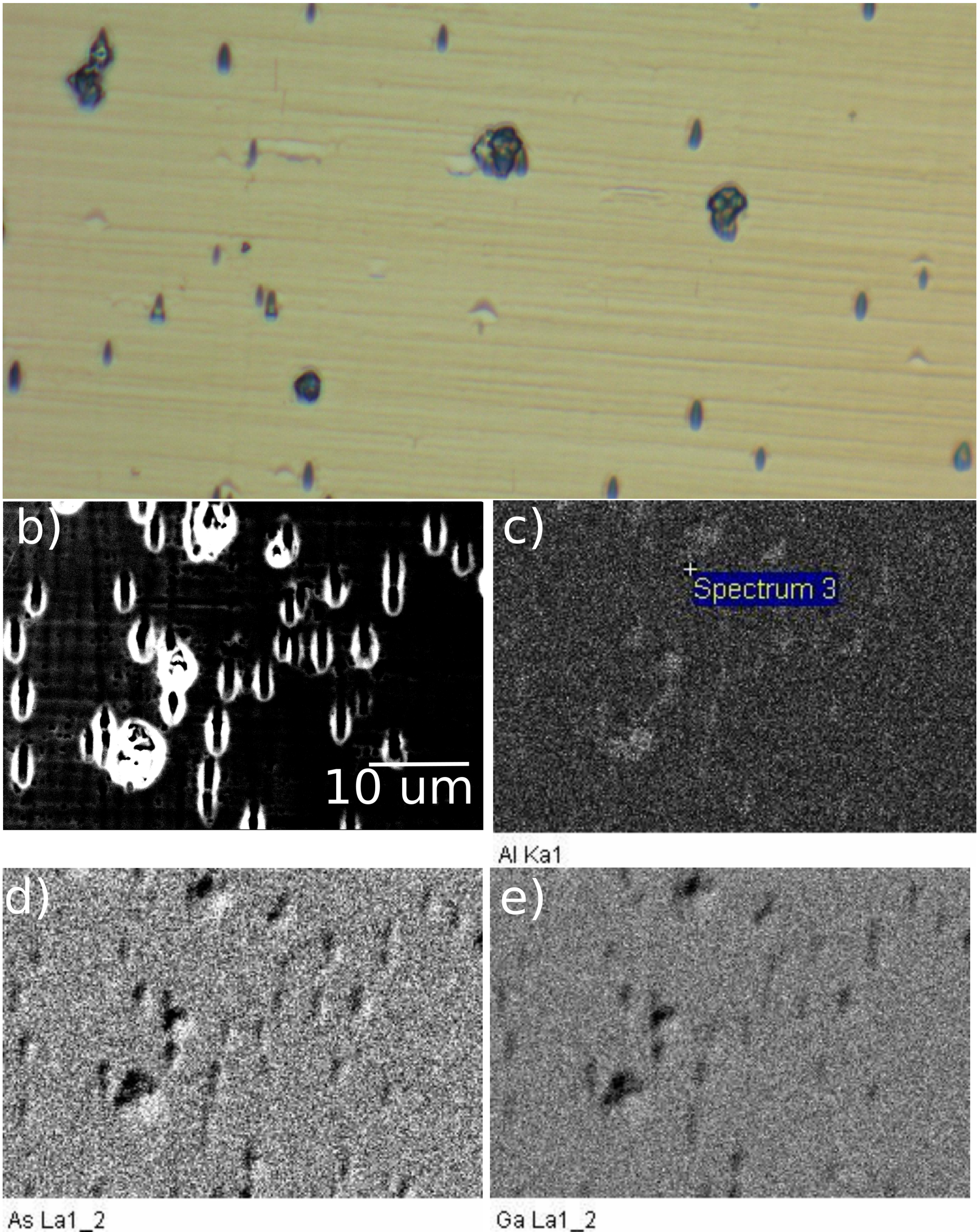

Figure S6: Top row: Image of C8 (1:1:10/55s) acquired using a light microscope at 50× magnification. The larger, more inhomogeneous structures are on top of the lighter surface, the parallel ellipsoidal structures are holes. b) Image of C8 (1:1:10/55s) acquired using a scanning electron microscope at 2500× magnification, 15 kV acceleration voltage, 10.1 mm working distance. c)-e) From the EDX mapping, the larger, more inhomogeneous structures consist of residual GaAsP on top of the AlInP surface. The parallel ellipsoidal structures are holes exposing GaAsP.

3.3.2 Volume Ratio 1:1:200

3.3.3 Volume Ratio 1:100:10 000

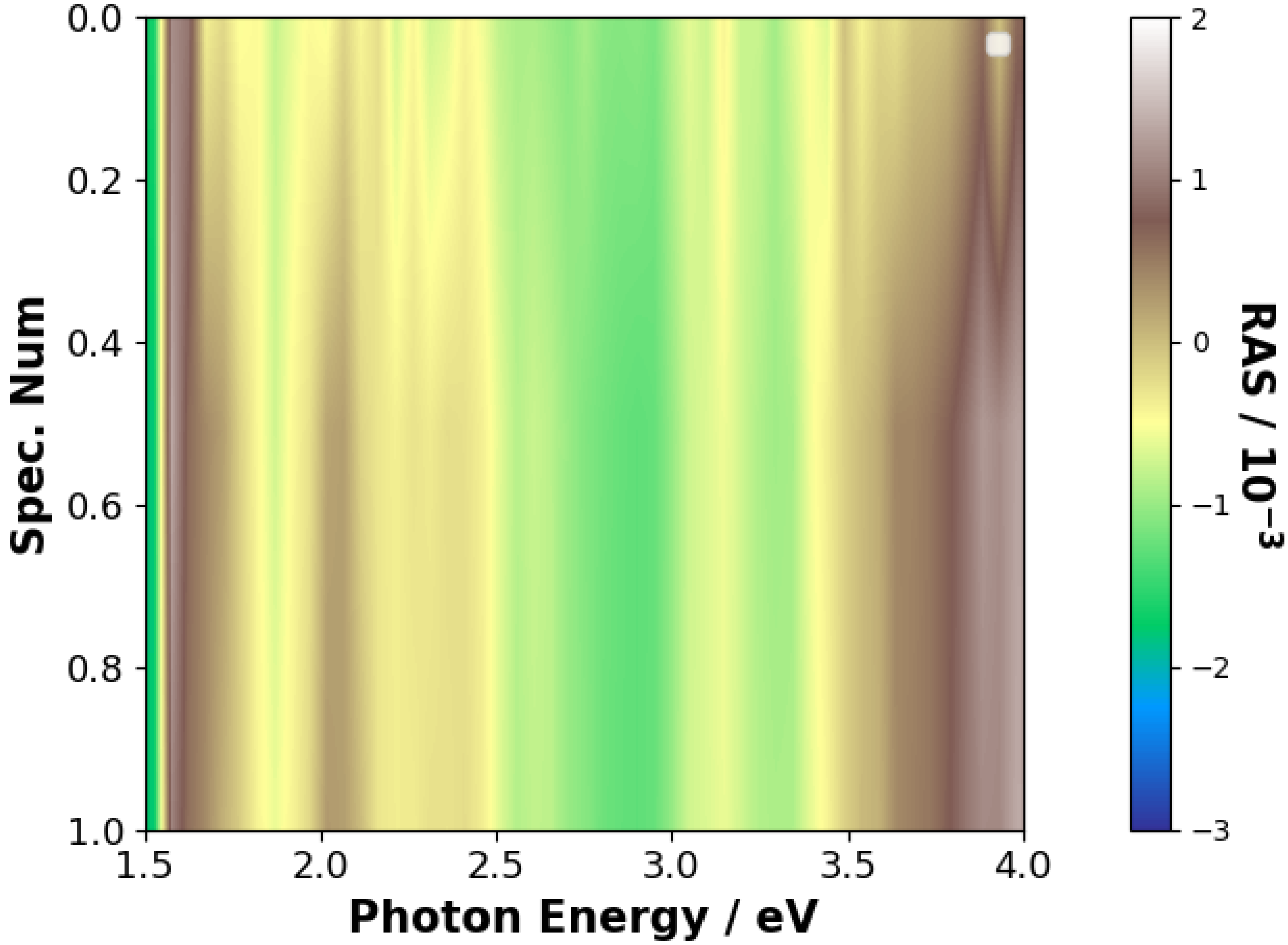

Figure S7: Colour plot of sample C4 during etching in a 1:10:10000 solution for 5648 s.

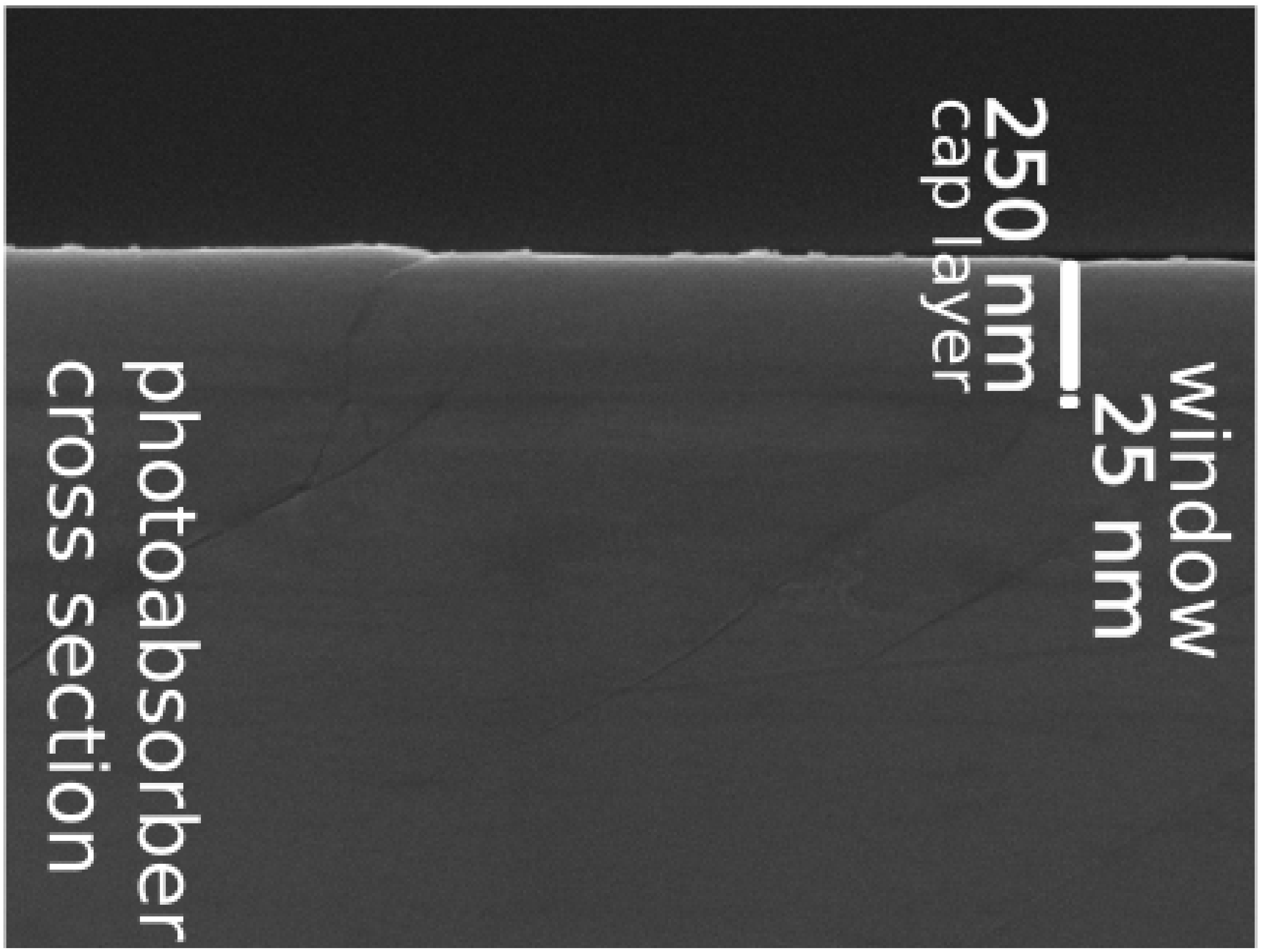

Figure S8: Cross section SEM of C4 showing the intact cap layer (250 nm) and below lying window layer (25 nm).

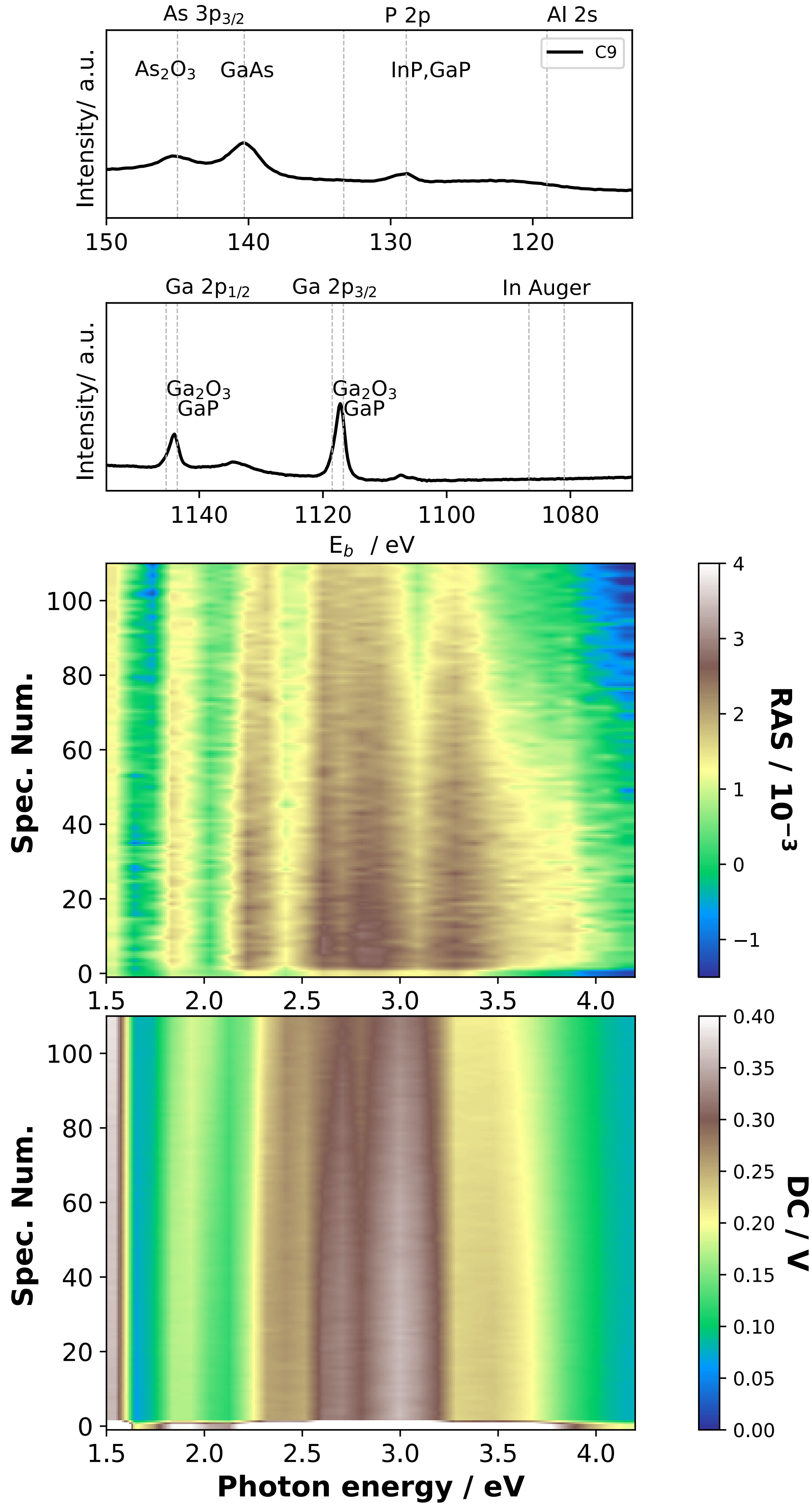

Figure S9: XPS and RAS of C9 (1:100:10 000/2820s).

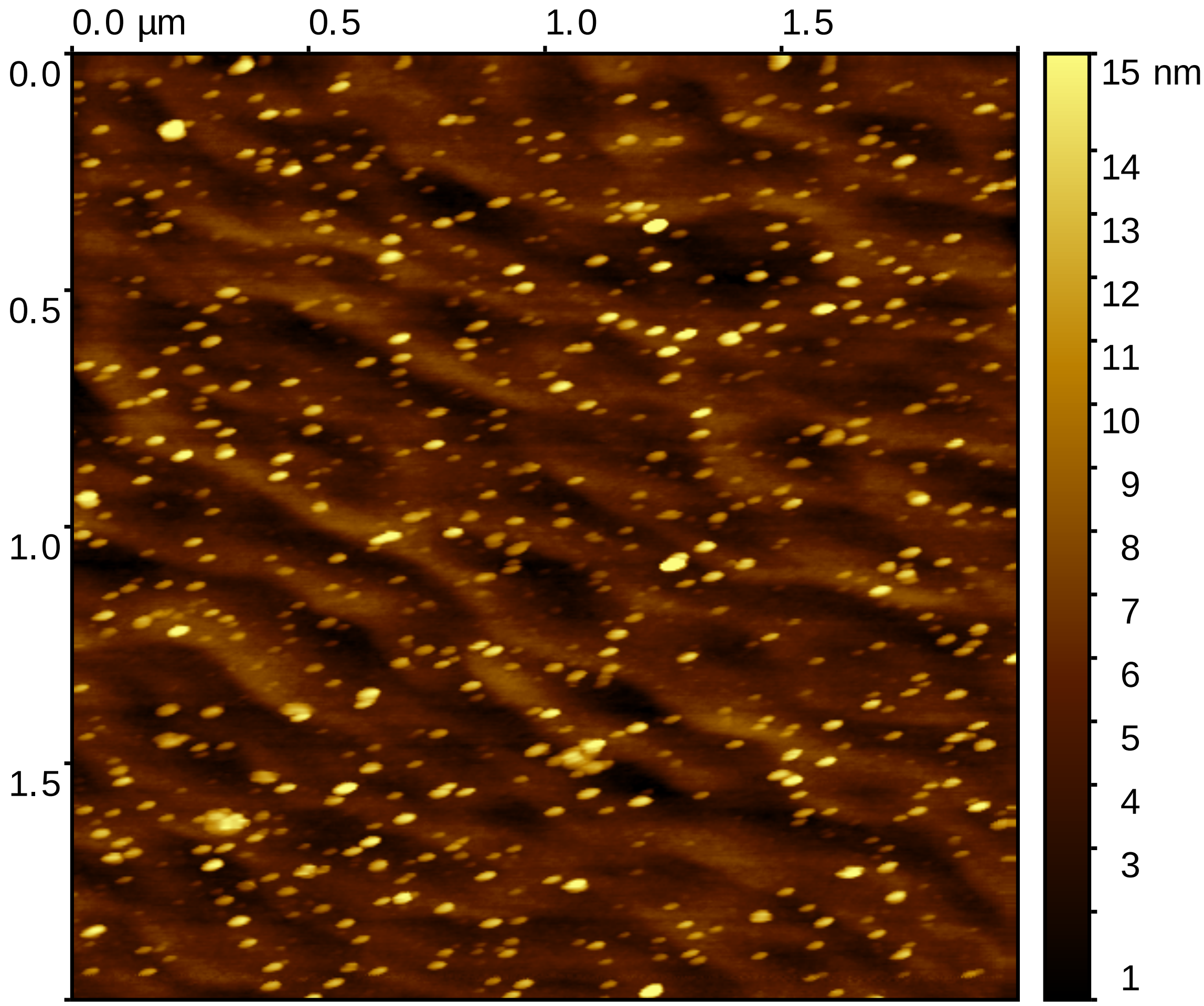

Figure S10: AFM of C4 (1:10:10000/5648s).

3.3.4 Additional Washing Step for Oxide Free Surface

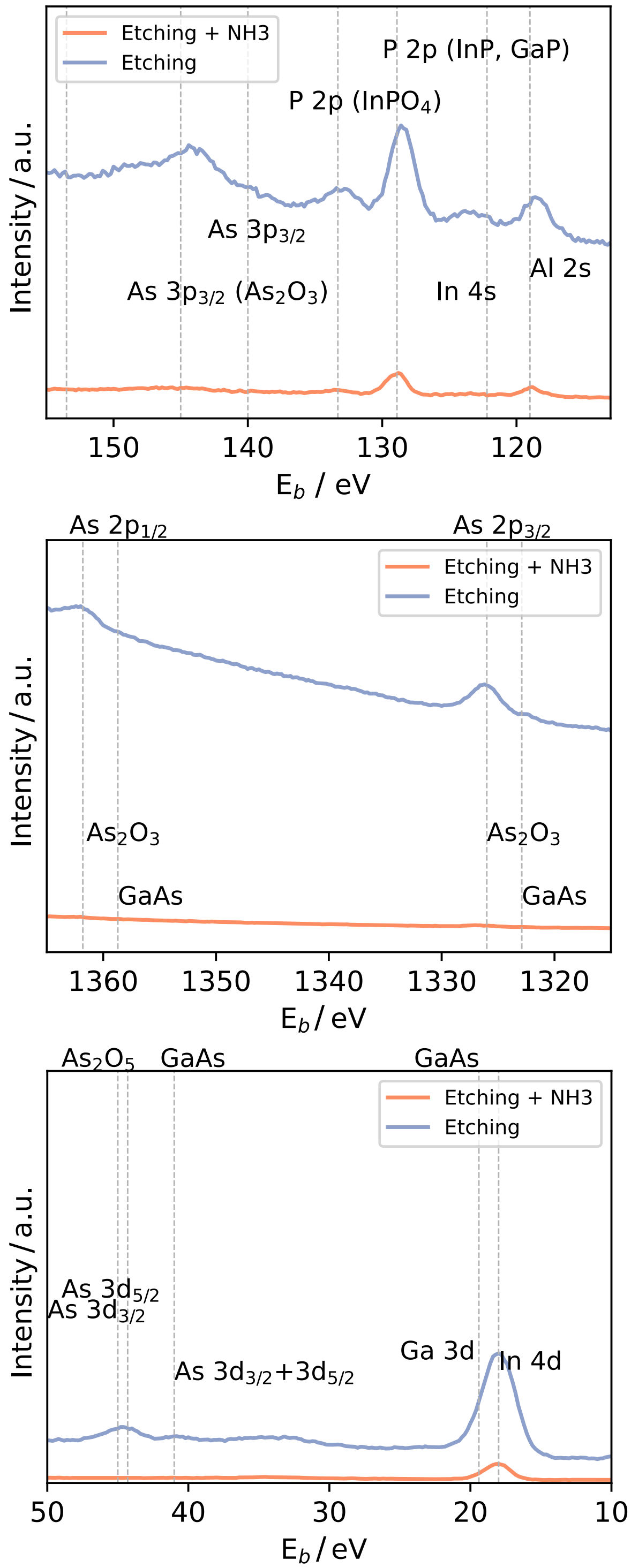

Figure S11: XPS measurements of the Samples AZUR Space I and II after removal of the 500 nm GaAs cap layer on top of a AlInP window layer. After etching, AZUR Space II is additionally washed for 60 s with a 3.3 mol/L solution of NH_3 (Sigma-Aldrich, p.a., 25%) in water (Milli-Q grade). The samples are taken from the same wafer produced by AZUR SPACE.

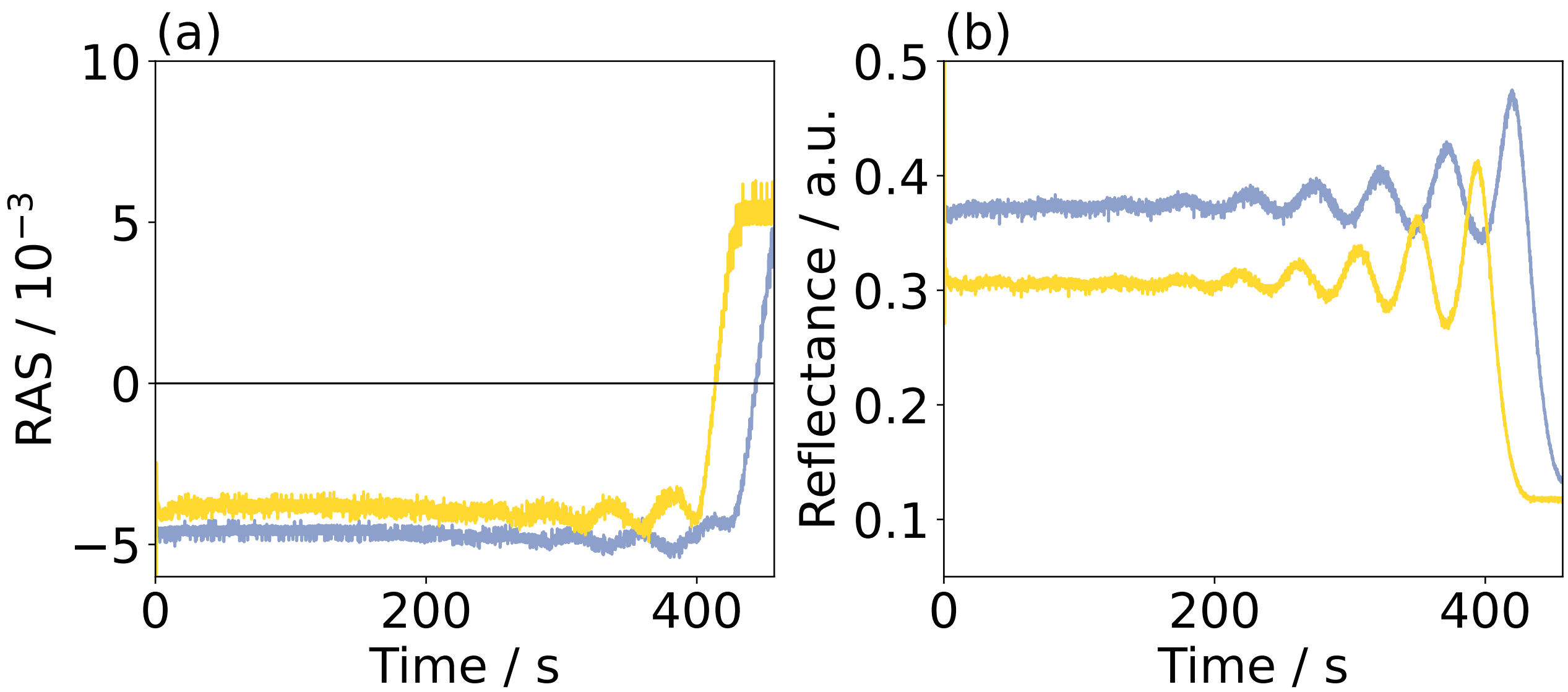

Figure S12: RAS and DC transients acquired at 2.6 eV with a volume ratio of the etching solution of 1:1:200. Samples A3 (blue) and A4 (yellow) with a 500 nm GaAs cap layer on top of a AlInP window layer are etched under the same conditions as samples A1 and A2. The samples are taken from the same wafer designed and produced by AZUR SPACE.

3.4 Deviations from oscillating behaviour due to initial structural constraints

Table S1: Roughness Parameters

Sample N°	C0	C3	C4	C1
RMS roughness (Sq) /nm	3.2	40.5	2.1	0.6
Mean roughness (Sa) /nm	2.5	32.9	1.4	0.5
Maximum height (Sz) /nm	33.0	365.1	24.8	7.3

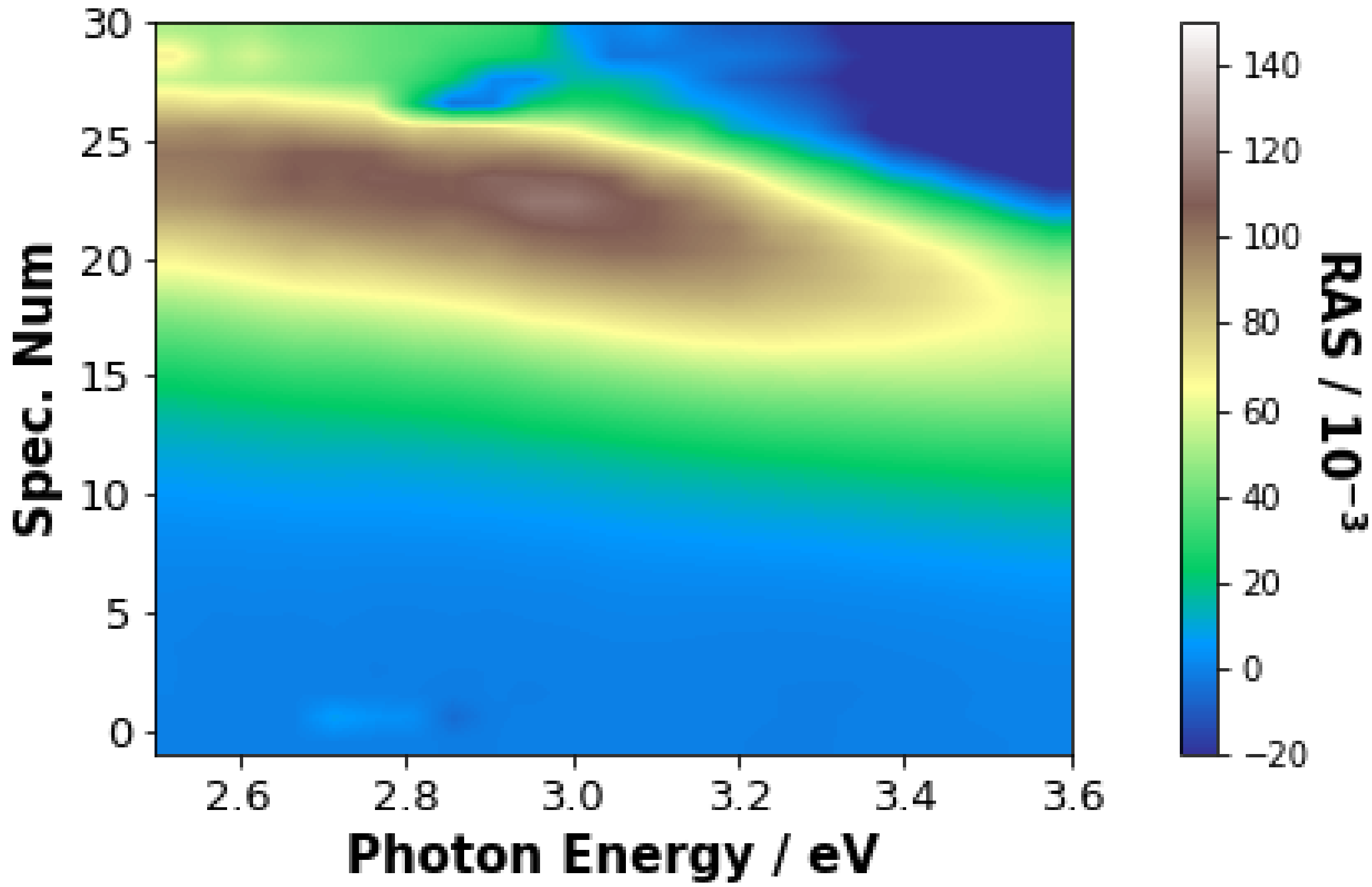

Figure S13: Colour plot of sample C3 during etching in a 1:1:200 solution for 400 s.

3.5 Analytical Methods

X-ray photoelectron spectroscopy (XPS) analyses were carried out by using a Specs Phoibos spectrometer with an Al K_α X-ray source. Survey scan analyses (0-1386 eV range) were carried out using a pass energy of 50 eV. Chemical state analyses were carried out by using a pass energy of 50 eV.

Atomic Force Microscopy measurements were carried out at a Bruker MultiMode 8HR system in peak force tapping mode at 1 nN force setpoint.

Scanning electronmicroscopy (SEM) measurements were carried out at a fJSM-6500F with a Schottky field emitter. Acceleration voltages of 0,5 kV - 30 kV and a max. resolution of 2 nm (5 - 30 kV) is possible.

List of Figures

S1 Self-designed PEEK sample holder with magnetic closing mechanism for glue free sample handling. S3

S2 Absorption coefficient over photon energy. S4

S3 Layer thickness of a GaAsP layer with error bar from uncertainty of n(λ). S5

S4 DC transients acquired upon etching of a) C5 at 2.6 eV (etching ratio 1:1:200), b) C6 at 2.0 eV (volume ratio 1:1:200) and c) C7 at 2.6 eV (etching ratio 1:1:10) d) C8 at 2.0 eV (etching ratio 1:1:10). The black curves correspond to the transient data filtered with a Savitzky-Golay filter (Filter: 10, polynomial: 3). The black dots localize the extrema of the oscillations. S7

S5 XPS spectra of C1 (1:1:200/540s) and C8 (1:1:10/30s). S8

S6 Top row: Image of C8 (1:1:10/55s) acquired using a light microscope at 50× magnification. The larger, more inhomogeneous structures are on top of the lighter surface, the parallel ellipsoidal structures are holes. b) Image of C8 (1:1:10/55s) acquired using a scanning electron microscope at 2500× magnification, 15 kV acceleration voltage, 10.1 mm working distance. c)-e) From the EDX mapping, the larger, more inhomogeneous structures consist of residual GaAsP on top of the AlInP surface. The parallel ellipsoidal structures are holes exposing GaAsP. S9

S7 Colour plot of sample C4 during etching in a 1:10:10000 solution for 5648 s. S10

S8 Cross section SEM of C4 showing the intact cap layer (250 nm) and below lying window layer (25 nm). S11

S9 XPS and RAS of C9 (1:100:10 000/2820s). S12

S10 AFM of C4 (1:10:10000/5648s). S13

S11 XPS measurements of the Samples AZUR Space I and II after removal of the 500 nm GaAs cap layer on top of a AlInP window layer. After etching, AZUR Space II is additionally washed for 60 s with a 3.3 mol/L solution of NH_3 (Sigma-Aldrich, p.a., 25%) in water (Milli-Q grade). The samples are taken from the same wafer produced by AZUR SPACE. S14

S12 RAS and DC transients acquired at 2.6 eV with a volume ratio of the etching solution of 1:1:200. Samples A3 (blue) and A4 (yellow) with a 500 nm GaAs cap layer on top of a AlInP window layer are etched under the same conditions as samples A1 and A2. The samples are taken from the same wafer designed and produced by AZUR SPACE. S15

S13 Colour plot of sample C3 during etching in a 1:1:200 solution for 400 s. S16

List of Tables

S1 Roughness Parameters S15
